# Antimicrobial resistance in community-acquired enteric pathogens among children aged ≤ 10-years in low-and middle-income countries: a systematic review and meta-analysis

**DOI:** 10.3389/fmicb.2025.1539160

**Published:** 2025-04-28

**Authors:** Noah O. Okumu, Dishon M. Muloi, Arshnee Moodley, Julie Watson, Alice Kiarie, Linnet Ochieng, Joseph O. Wasonga, Christine Mutisya, Lorren Alumasa, Joseph J. N. Ngeranwa, Oliver Cumming, Elizabeth A. J. Cook

**Affiliations:** ^1^Health Program, International Livestock Research Institute, Nairobi, Kenya; ^2^Department of Biochemistry, Biotechnology and Microbiology, Kenyatta University, Nairobi, Kenya; ^3^Institute of Infection, Veterinary and Ecological Sciences, University of Liverpool, Liverpool, United Kingdom; ^4^Department of Veterinary and Animal Sciences, University of Copenhagen, Frederiksberg C, Denmark; ^5^Department of Disease Control, London School of Hygiene and Tropical Medicine, London, United Kingdom

**Keywords:** antimicrobial resistance, low-and middle-income countries, enteric bacteria, community, children

## Abstract

**Introduction:**

Antimicrobial resistance (AMR) is a global health priority. This systematic review summarizes the prevalence of AMR in enteric pathogens originating from the community, specifically among ≤10-year-old children in low-and middle-income countries (LMICs). In addition, it presents the proportions of pooled resistance in *Campylobacter* spp., *Escherichia coli*, *Shigella* spp., and *Salmonella* spp. (CESS) to clinically relevant antibiotics.

**Methods:**

Six online repositories, namely PubMed, Medline, Web of Science, Cochrane Library, CABI, and EMBASE were searched for articles published between January 2005 and September 2024. Random-effects meta-analysis models were constructed to estimate the pooled AMR proportions for CESS pathogens, and a subgroup analysis by region was also carried out.

**Results:**

A total of 64 publications from 23 LMICs met our inclusion criteria. The pooled estimates of *E. coli* AMR for clinically important antibiotics were as follows: sulfamethoxazole/trimethoprim (SXT) 71% [95%CI: 57–82%]; ampicillin (AMP) 56% [95%CI: 44–67%]; ciprofloxacin (CIP) 10% [95%CI: 5–20%]; and ceftriaxone (CRO) 8% [95%CI: 2–31%]. The proportions of AMR detected in *Shigella* spp. were AMP 76% [95%CI: 60–87%]; nalidixic acid (NA) 9% [95%CI: 2–31%]; CIP 3% [95%CI: 0–15%]; and CRO 2% [95%CI: 0–19%]. The proportions of *Salmonella* spp. AMR were AMP 55% [95%CI: 35–73%] and SXT 25% [95%CI: 15–38%]. The proportions of *Campylobacter* spp. AMR were erythromycin (ERY) 33% [95%CI: 12–64%] and CIP 27% [95%CI: 8–61%]. There was high variability in the regional subgroup analysis, with high interstudy and regional heterogeneity *I^2^* ≥ 75%.

**Conclusion:**

Our results shed light on drug-resistant enteric bacterial pathogens in young children, providing evidence that CESS pathogens are becoming increasingly resistant to clinically important antimicrobials. Regional differences in resistance patterns between these community isolates highlight the need for strong national and regional surveillance to detect regional variations and inform treatment and appropriate antibiotic stewardship programs. The limitations of our findings include high regional variability, significant interstudy heterogeneity, and underrepresentation of certain LMICs.

**Systematic review registration:**

https://inplasy.com/inplasy-2024-2-0051/, registration number: INPLASY202420051.

## 1 Introduction

Antimicrobial resistance (AMR) is a significant threat to global health, contributing to an estimated 1.27 million deaths worldwide in 2019, with the highest mortality rates reported in sub-Saharan Africa and South Asia (Barber and Sutherland, 2017; Murray et al., 2022). AMR aggravates health complications and increases the risk of mortality and economic burden undermining childhood survival in low- and middle-income countries (LMICs) (Huynh et al., 2015; Sulis et al., 2022). LMICs are often characterized by limited access to healthcare, inadequate sanitation and hygiene, and a high burden of infectious diseases, such as pneumonia, diarrhea, and malaria (Barber and Sutherland, 2017; Hendriksen et al., 2019), driving both prophylactic and therapeutic antimicrobial use (Omulo et al., 2015).

A key factor in addressing the challenge of AMR is surveillance (World Health Organization, 2017a). Community AMR surveillance involves monitoring the prevalence and patterns of AMR in community to facilitate early detection of resistant strains and allow timely interventions to prevent further spread (Kasolo et al., 2013). However, comprehensive population-based AMR surveillance data are lacking in both LMICs and high-income countries (HICs), thus casting doubt on the accuracy of global estimates of AMR burden (Gandra et al., 2020). LMICs that have developed national action plans for AMR often rely on fragmented and non-representative surveillance data from larger urban hospitals, with obvious gaps in human population- or community-based AMR data; thus, their usefulness to healthcare policymakers is limited (Otto et al., 2022; Iskandar et al., 2021). High-quality AMR surveillance data help monitor treatment guidelines by assessing the effectiveness of current recommendations and making necessary adjustments. Through surveillance, high-risk areas or vulnerable populations such as children can be identified in order to guide the implementation of infection control to prevent the spread of resistant strains.

Variability has been reported in the capability to conduct AMR surveillance in low-resource settings due to challenges such as weak laboratory infrastructure, limited resources, overreliance on donor funding, and a lack of qualified staff and training (Iskandar et al., 2021). The Global Antimicrobial Resistance Surveillance System (GLASS) has endeavored to support evidence-based and standardized surveillance worldwide (Gandra et al., 2020). Following this GLASS initiative, national AMR surveillance systems have been established in some South and Southeast Asian countries, including Bangladesh, Cambodia, India, Laos, Nepal, Pakistan, Thailand, and Vietnam (Gandra et al., 2020; World Health Organization, 2017b; Aggarwal et al., 2012; World Health Organization, 2016). Although GLASS initiatives support standardized global surveillance, reports on community circulation of bacteria with AMR remain limited, particularly in Africa, where only 23 of 54 countries have implemented national AMR systems (Okolie et al., 2022).

Previous reviews have focused on the burden associated with AMR in healthcare-acquired infections (HAIs), reporting a higher burden in LMICs than in HICs (Ayobami et al., 2022; Allegranzi et al., 2011). However, there is a paucity of comprehensive data on the prevalence and distribution of AMR in community-acquired enteric bacteria, especially in vulnerable ≤10-year-old children in resource-limited settings. Enteric pathogens, namely *E. coli*, *Salmonella*, *Shigella*, and *Campylobacter*, were investigated in this review since they are among the predominant bacterial causes of diarrhea in children (CDC, 2022; Guarino et al., 2018). Diarrhea remains the second leading cause of infant mortality, preceded by pneumonia (Liu et al., 2012). In LMICs, inappropriate use of antimicrobials to manage diarrhea and other childhood diseases in community settings contributes to AMR (Farthing et al., 2013). Antimicrobial use not only alters the gut microbiome but also selects for bacteria with AMR, turning innocuous gut commensals into reservoirs of AMR determinants that can be transmitted to pathogens or across epidemiological compartments through prolonged fecal shedding (World Health Organization, 2005b). Due to its abundance in the gut, ease of isolation, widespread distribution, and significant genetic diversity, *E. coli* has been used as an indicator of overall AMR patterns within community, providing insights into the prevalence and distribution of resistance.

Thus, this review addresses critical gaps in the literature by estimating pooled proportions of AMR in clinically important, community-acquired enteric bacteria in ≤10-year-old children across LMICs. By analyzing resistance patterns across geographical regions, we aim to identify the gaps in AMR surveillance and highlight areas where action is required to mitigate the burden of AMR in resource-limited settings.

## 2 Methods

**Figure 1:**
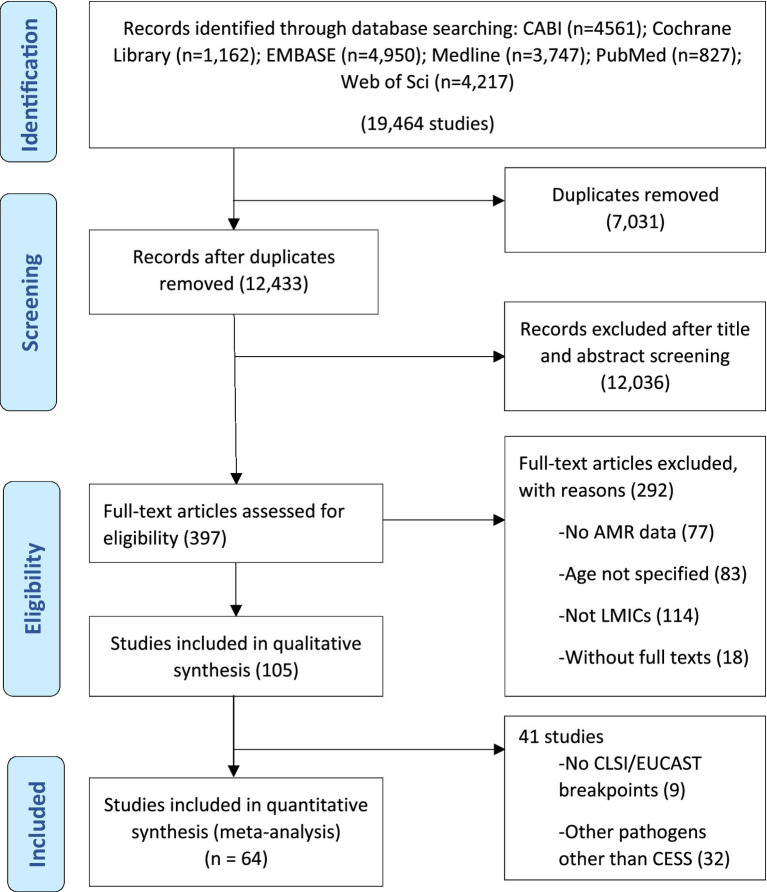
Systematic selection for inclusion of studies on community-acquired antimicrobial resistance in children in low- and middle-income countries.

This systematic review and meta-analysis was conducted in accordance with the Preferred Reporting Items for Systematic Reviews and Meta-analyses (PRISMA) guidelines (Figure 1) and in compliance with the PRISMA checklist (Supplementary Table 1) (Shamseer et al., 2015). The study protocol was registered on INPLASY (INPLASY202420051) to ensure full compliance with best practices in research transparency and protocol registration.

### 2.1 Search strategy and selection criteria

As defined by the World Bank, various combinations of search terms related to child descriptors, AMR, and LMICs were used to search numerous databases, including PubMed, Medline, Web of Science, Cochrane Library, CABI, and EMBASE (World Bank, 2019). The initial database searches were conducted between July 14 and August 5, 2020, and revised searches were performed between September 20 and September 22, 2024. The complete search strategy is provided in Supplementary File 1. Articles were screened for eligibility using the following inclusion criteria: (1) original articles published or accepted in a peer-reviewed journal between January 2005 and September 2024; (2) participants (≤10-year-old children) residing in an LMIC; (3) cross-sectional or longitudinal design with baseline data; and (4) having measured AMR in bacterial isolates obtained from children in non-clinical or outpatient settings or from samples collected within 48 h of hospital admission, to minimize the inclusion of healthcare-acquired infections. The year 2005 was purposefully selected because it was the year the World Health Organization began emphasizing AMR as a critical public health challenge (World Health Organization, 2005b). Studies published since 2005 are more likely to reflect contemporary diagnostic, treatment, and surveillance practices, making them more relevant to our research objectives. Including data up to September 2024 ensured that the review captures the most up-to-date evidence, reflecting the current status of AMR in community-acquired enteric pathogens, particularly in LMICs. Case reports and review papers were excluded. Non-English articles were translated using Google Translate, and key sections (Methods and Results) were cross-checked for accuracy. The identified articles were shared among four co-authors (NO, LO, AK, and LA) who independently screened the titles and abstracts and reviewed the full texts of potentially relevant articles. Disagreements between the reviewers were resolved through consensus.

### 2.2 Data extraction

Data were extracted by the same four independent reviewers (NO, LO, AK, and LA) using a predefined and standardized data extraction form. The extracted information included (1) year of publication, (2) first author’s name, (3) study location (country/region), (4) study setting (hospital, community, or both), (5) year of sample collection, (6) participant demographics (age and sex) and health status, (7) sample size, (8) sample type, (9) bacterial species isolated, (10) number of isolates, (11) study design, (12) antimicrobial susceptibility testing (AST) method, and (13) prevalence of AMR (i.e., the number of resistant isolates). The quality of the articles included in the review was assessed using the Newcastle Ottawa Quality Assessment Scale (NOS) adapted for cross-sectional study design (Feeley, 2020). This assessment was conducted using three parameters: the selection of the sample, the comparability of the tests, and the level of exposure. The selection parameter was based on the representativeness of the sample, the definition of the community, and the sample size. The comparability parameter was based on the bacterial isolation and identification method and the AST method. The exposure parameter was based on the number of AMR isolates. A study with an overall score of ≥7 out of 10 was considered to be of good quality and was included in the analysis to obtain a revised pooled estimate.

### 2.3 Statistical analysis

The primary outcome of our analysis was the proportion of bacterial isolates that showed resistance to an antimicrobial. We examined the prevalence of AMR in different enteric bacterial species, such as *E. coli*, *Shigella* spp., *Salmonella* spp., *Campylobacter* spp., *Vibrio cholerae*, and *Enterococcus* spp. However, the meta-analysis focused on the four most reported enteric bacterial pathogens: *Campylobacter* spp., *E. coli*, *Shigella* spp., and *Salmonella* spp. (hereafter referred to as CESS). The isolate–drug combinations included drugs commonly reported in the articles and indicated for first- or second-line empiric treatment. These drugs included ciprofloxacin (CIP), gentamicin (CN), sulfamethoxazole/trimethoprim (SXT), ceftriaxone (CRO), ampicillin (AMP), amoxicillin/clavulanate (AMC), nalidixic acid (NA), azithromycin (AZM), erythromycin (ERY), and tetracycline (TET).

All data analyses were performed using R (version 4.2.2). Meta-analyses of the proportions of the tested bacteria resistant to each antimicrobial were conducted using the “metaprop” function of the “dmetar” and “meta” packages. Meta-analyses were stratified by isolate–drug combination and geographical region (*n* ≥ 2 studies). Due to the high levels of heterogeneity (*I^2^* > 75%) between studies, a random-effects model was used to pool effect sizes (i.e., pooled proportion of pathogens resistant to a particular antimicrobial). Random-effects meta-analysis is a statistical approach that accounts for variability between studies and provides a more generalized effect estimate across different populations. AMR proportions were pooled using a generalized linear mixed-effects model (GLMM) with logit-transformed proportions, using the following equation:

(1) $${\hat{p}}_{\mathrm{pool}}=\frac{\sum_{i=1}^{k}\left(w_{i}.{\hat{p}}_{i}\right)}{\sum_{i=1}^{k}w_{i}}$$

where:

${\hat{p}}_{\mathrm{pool}}$ represents the pooled proportion estimate, which is the combined estimate of the proportion of the resistance of *E. coli* to an antibiotic (e.g., AMP) across all included studies;

k refers to the number of studies included in the meta-analysis, each study contributing an estimate of the proportion of resistance;

${\hat{p}}_{i}$ is the estimated proportion of the resistance of *E. coli* to AMP in study i . It is calculated by dividing the number of resistant isolates by the total number of isolates in study i ; and.

$w_{i}$ represents the weight assigned to study i . In GLMM, weights are derived from the estimated variances of the proportion estimates, considering both within-study and between-study variability.

The GLMM incorporates both fixed and random effects to model the variability in proportion estimates across studies. It allows for the estimation of study-specific effects (fixed effects) and the overall pooled effect (random effects). Using Equation 1, a weighted sum of the individual study proportion estimates is calculated, where each estimate is weighted by its corresponding weight $w_{i}$ . The weights are derived from the GLMM framework, which accounts for the heterogeneity between studies. The denominator is the sum of weights, ensuring that ${\hat{p}}_{\mathrm{pool}}$ is appropriately scaled.

Variability between studies was assessed through heterogeneity tests using the *I^2^* statistic, which was calculated using the “dmetar” package (Smellie, 2006). An *I*^2^ index greater than 50% indicated moderate to substantial heterogeneity (Higgins and Thompson, 2002). Influence analysis was conducted to identify the impact of outliers on the overall effect estimate, following the approach outlined by Viechtbauer et al. (2015). Outliers have extremely small and large effects that significantly differ from the overall effect based on 95% CI. To find and remove outlier studies and to measure the pooled effect size without them, the *“find.outliers”* function was used. Furthermore, Egger’s regression test for funnel plot asymmetry was performed to measure publication bias using the following equation:

(2) $$\frac{{\hat{\theta }}_{k}}{SE_{{\hat{\theta }}_{k}}}=\beta_{0}+\beta_{1}\frac{1}{SE_{{\hat{\theta }}_{k}}}$$

where the response, y , is the observed effect size ${\hat{\theta }}_{k}$ divided by the standard error, $SE_{{\hat{\theta }}_{k}}\text{,}$ which gives z -scores. In this test (Equation 2), the intercept $\left(\beta_{0}\right)$ rather than the regression weight ( $\beta_{1})$ is used to evaluate the funnel plot asymmetry. If the size of ${\hat{\beta }}_{0}$ is significantly different from zero, then the test shows funnel plot asymmetry, i.e., presence of publication bias (Harrer et al., 2021).

## 3 Results

**Figure 2:**
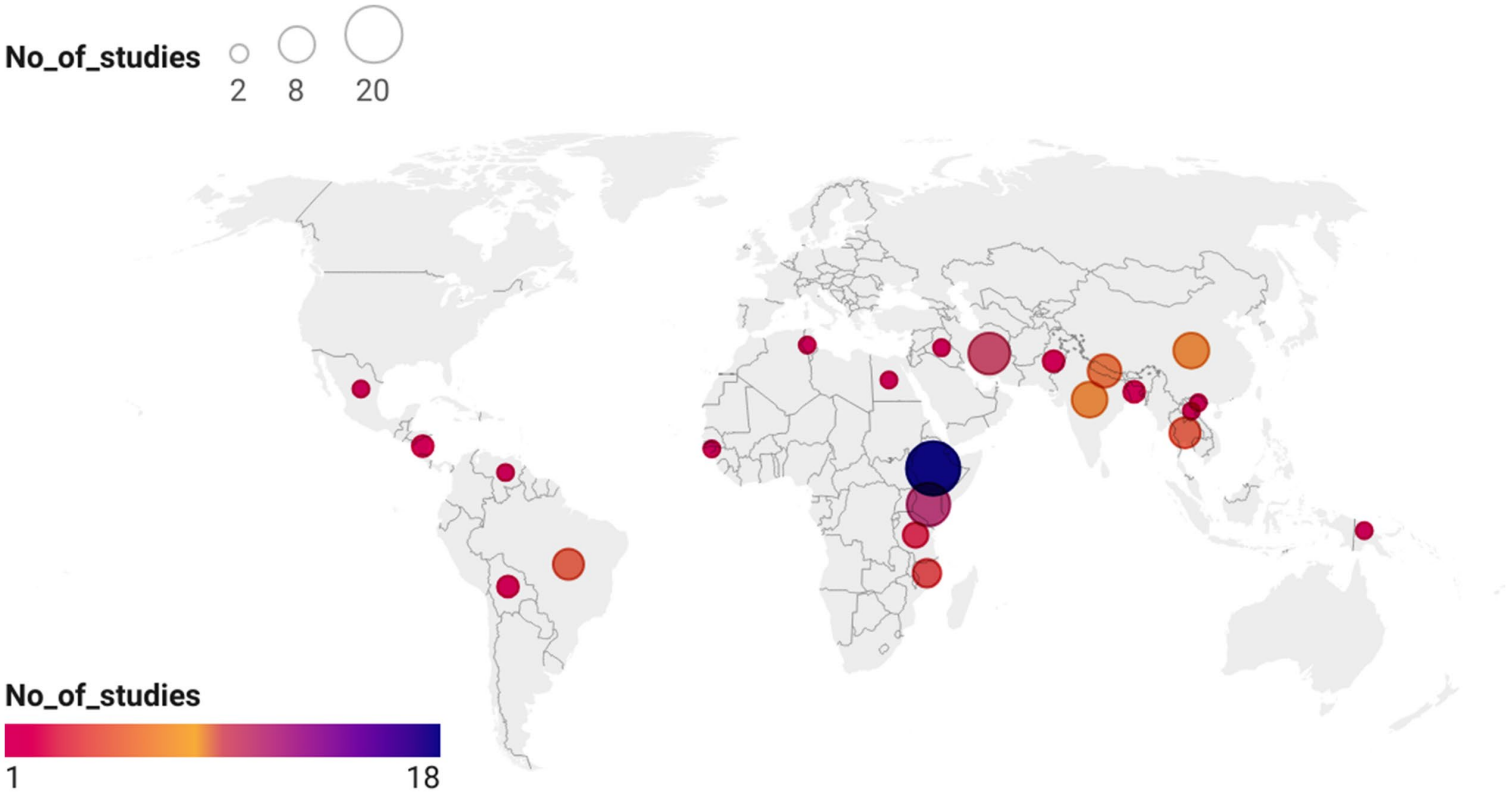
Locations by the country of 64 publications included in this systematic review. The basemap of the world was created using datawrapper: https://datawrapper.dwcdn.net/p5txr/1/. The basemap shape file for the world was downloaded from https://www.datawrapper.de/maps/choropleth-map.

Electronic searches yielded 19,464 publications, which was reduced to 12,433 after the removal of duplicates (Figure 1). After screening and exclusion, 105 publications met our inclusion criteria. Out of these 105 publications, 64 provided data on the four most commonly reported enteric bacteria (*E. coli*, *Shigella*, *Salmonella*, and *Campylobacter*) and were included in the meta-analyses (Figure 1). Each individual bacterial species reported in these 64 articles was treated as a unique study. Therefore, this resulted in a total of 93 studies (representing 8,082 isolates). Among these 93 studies, 44 focused on *E. coli* (*n* = 5,941 isolates), 23 on *Shigella* (*n* = 836 isolates), 18 on *Salmonella* (*n* = 690 isolates), and 8 on *Campylobacter* (*n* = 615 isolates). Sub-Saharan Africa had the highest number of eligible studies (*n* = 37), followed by Asia (*n* = 31), North Africa/Middle East (*n* = 14), Central and South America (*n* = 10), and North America (*n* = 1). Ethiopia had the highest number of eligible studies (*n* = 18), followed by Kenya (*n* = 11) and Iran (*n* = 10) (Figure 2). A summary of the characteristics of the included studies is presented in Table 1.

**Table 1:** Characteristics of 64 articles included in the meta-analysis of community-acquired AMR in under-10-year-old children in low- and middle-income countries.

Author	Country	Geographical region	Study setting	Study period	Study design	Age	Gender	*Health status	Sample size	Sample type	Bacterial species	No. of isolates	AST interpretive criteria
Saleem et al. (2020)	Pakistan	Asia	Community		Cross-sectional	0–5 yrs	58 m;46f	Healthy	104	Stool	*E. coli*	62	CLSI
Snehaa et al. (2020)	India	Asia	Hospital	Jun 2014-Jun 2015	Cross-sectional	0–5 yrs		Clinical	200	Stool	*E. coli*	19	CLSI
Webale et al. (2020)	Kenya	Sub-Saharan Africa	Hospital		Cross-sectional	0–5 yrs	193 m; 181F	Clinical	374	Stool	*E. coli, Campylobacter, Salmonella, Shigella*	136	CLSI
Araque and Labrador (2018)	Venezuela	Central/South America	Community	Jan-Jul 2015	Cross-sectional	0–5 yrs	42 m; 36f	Healthy	78	Stool	*E. coli*	78	CLSI
Al-saadi et al. (2018)	Iraq	North Africa/Middle East	Hospital	Jul 2016–Feb 2017	Cross-sectional	0–5 yrs	289 m;211f	Clinical	500	Stool	*E. coli*	11	CLSI
Singh et al. (2019)	India	Asia	Hosp/Community	Jul 2013-Jul 2015	Cross-sectional	0–5 yrs	75 m;45f	Clinical/healthy	120	Stool	*E. coli*	120	CLSI
Zheng et al. (2016)	China	Asia	Hospital	Jul 2009–Dec 2014	Cross-sectional	0–5 yrs		Clinical	2,318	Stool	*E. coli*	177	CLSI
Jafari et al. (2009)	Iran	North Africa/Middle East	Hospital	May 2003–May 2005	Cross-sectional	0–5 yrs	613 m;507f	Clinical	1,120	Stool	*E. coli, Shigella*	35	CLSI
Seidman et al. (2009)	India	Asia	Community	Nov 2005-Jan 2006	Cross-sectional	0–10 yrs	54 m;66f	Healthy	120	Stool	*E. coli*	119	CLSI
Mandomando et al. (2007)	Mozambique	Sub-Saharan Africa	Hospital	Sep 2000-Sep 2001	Cross-sectional	0–5 yrs	294 m;235f	Clinical	529	Stool	*E. coli, Salmonella*	94	CLSI
Islam et al. (2019)	Bangladesh	Asia		Mar -Oct 2017	Cross-sectional	0–5 yrs		Clinical	100	Stool	*E. coli*	82	CLSI
Amin et al. (2018)	Iran	North Africa/Middle East	Hospital	Mar 2015 -Feb 2016	Cross-sectional	0–10 yrs			255	Stool	*E. coli*	32	CLSI
Adugna et al. (2015)	Ethiopia	Sub-Saharan Africa	Hospital	Dec 2011 – Feb 2012	Cross-sectional	0–5 yrs	239 m;183F	Clinical	422	Stool	*E. coli*	204	CLSI
Moyo et al. (2011)	Tanzania	Sub-Saharan Africa	Hospital	Dec 2005 -Feb 2006	Cross-sectional	0–5 yrs	172 m;108f	Clinical	280	Stool	*E. coli, Salmonella, Shigella*	64	CLSI
Gebresilasie et al. (2018)	Ethiopia	Sub-Saharan Africa	Hospital	Aug -Dec 2015	Cross-sectional	0–5 yrs		Clinical	253	Stool	*E. coli*	64	CLSI
Mekonnen et al. (2017)	Ethiopia	Sub-Saharan Africa	Hospital	Dec 2014–Mar 2015	Cross-sectional	0–5 yrs	105 m;91f	Clinical	196	Stool/rectal swabs	*E. coli, Salmonella, Shigella*	25	CLSI
Mahmoudi-aznaveh et al. (2017)	Iran	North Africa/Middle East	Hospital	Nov 2012 – Oct 2013	Cross-sectional	0–5 yrs		Clinical	547	Stool	*E. coli*	30	CLSI
Younas et al. (2016)	Pakistan	Asia	Community	2010–2012	Cross-sectional	0–5 yrs		Clinical	225		*E. coli*	46	CLSI
Stoesser et al. (2014)	Lao people’s Democratic Republic	Asia	Community	March–June 2011	Cross-sectional	0–10 yrs		Healthy	397	Stool	*E. coli*	78	CLSI
Vilchez et al. (2014)	Nicaragua	Central/South America	Hosp/Community	Jan 2010 – Jan 2011	Cross-sectional	0–5 yrs		Clinical	720	Stool	*E. coli*	58	CLSI
Ansari et al. (2012)	Nepal	Asia	Hospital	Apr 2011 -Sept 2011	Cross-sectional	0–5 yrs		Clinical	525	Stool	*E. coli, Salmonella, Shigella*	12	CLSI
Giani et al. (2018)	Bolivia	Central/South America	Community	Sept-Oct 2016	Cross-sectional	0–10 yrs	158;179f	Healthy	337	Stool	*E. coli*	171	EUCAST
Monira et al. (2017)	Bangladesh	Asia	Community	Feb 2011 – Jul 2012	Cross-sectional	0–5 yrs		Healthy	15	Stool	*E. coli*	63	CLSI
Naderi et al. (2016)	Iran	North Africa/Middle East	Hospital		Case/control	0–5 yrs	364 m;236f	Clinical/healthy	600	Stool	*E. coli*	136	CLSI
Dias et al. (2016)	Brazil	Central/South America	Hosp/Community	Mar 2013-Sept 2014	Case/control	0–5 yrs	221 m;179f	Clinical/healthy	400	Rectal swabs	*E. coli*	38	CLSI
Bodhidatta et al. (2010)	Thailand	Asia	Hospital	Oct 2001-Oct 2002	Case/control	0–5 yrs	233 m;239f	Clinical/healthy	472	Stool	*E. coli, Campylobacter, Salmonella, Shigella*	44	CLSI
Gonzales et al. (2013)	Bolivia	Central/South America	Hospital	Jan 2007-Dec 2010	Case/control	0–5 yrs		Clinical	3,943	Stool	*E. coli*	881	CLSI
HaiLing et al. (2017)	China	Asia	Hosp/Community	2014	Case/control	0–5 yrs		Clinical/healthy	680	Stool	*E. coli, Campylobacter, Salmonella*	201	CLSI
Garcia et al. (2011)	Brazil	Central/South America	Hosp/Community	May 2007-Dec 2008	Case/control	0–5 yrs		Clinical/healthy	141	Stool	*E. coli*	136	CLSI
El Metwally et al. (2007)	Egypt	North Africa/Middle East	Hosp/Community	Mar-Dec 2005	Case/control	0–5 yrs		Clinical/healthy	200	Stool	*E. coli*	25	CLSI
Souza et al. (2009)	Brazil	Central/South America	Community	Aug 2007-Oct 2007	Cross-sectional	0–10 yrs		Healthy	114	Stool	*E. coli*	52	CLSI
Hetzer et al. (2019)	Thailand	Asia	Hosp/Community	Sept 2010-Dec 2012	Prospective	0–5 yrs		Healthy	142	Meconium/stool	*E. coli*	47	EUCAST
Singh et al. (2019)	India	Asia	Hosp/Community		Case/control	0–5 yrs	53 m;27f	Clinical/healthy	80	Stool	*E. coli*	55	CLSI
Anvikar et al. (2008)	India	Asia	Community	May 2004-Apr 2005	cohort	0–10 yrs		Clinical	580	Stool	*E. coli*	64	CLSI
Ferjani et al. (2018)	Tunisia	North Africa/Middle East	Community	2012–2013	Cross-sectional	0–10 yrs		Clinical	105		*E. coli*	98	CLSI
Mahdavi Broujerdi et al. (2018)	Iran	North Africa/Middle East	Hospital	Sept 2015–Jun 2016	Cross-sectional	0–5 yrs		Clinical	208	Stool	*E. coli*	54	CLSI
Huang et al. (2015)	China	Asia	Hospital	2009	Cross-sectional	0–5 yrs		Clinical	1,634	Stool	*E. coli*	58	CLSI
Nakhjavani et al. (2013)	Iran	North Africa/Middle East	Hospital	2009–2010	Cross-sectional	0–10 yrs		Clinical	612	Stool	*E. coli*	412	CLSI
Sang et al. (2012)	Kenya	Sub-Saharan Africa	Community	Oct 2007 – Sept 2008	Cross-sectional	0–5 yrs	349 m;302f	Clinical	651		*E. coli, Salmonella, Shigella*	73	CLSI
Dyar et al. (2012)	Vietnam	Asia		Mar -Jun 2007	Cross-sectional	0–5 yrs		Clinical/healthy	818	Stool	*E. coli*	738	CLSI
Isendahl et al. (2012)	Guinea-Bissau	Sub-Saharan Africa	Hospital	Jun -Sep 2010	Cross-sectional	0–5 yrs		Clinical	447	Rectal swabs	*E. coli*	83	EUCAST
Najibi et al. (2012)	Iran	North Africa/Middle East	Hospital	Apr-May 2009	Cross-sectional	0–5 yrs		Clinical	309	Stool	*E. coli*	29	CLSI
Sahoo et al. (2012)	India	Asia	Community		Cross-sectional	0–10 yrs		Healthy	1,251	Stool	*E. coli*	696	CLSI
Amaya et al. (2011)	Nicaragua	Central/South America		Mar 2005 – Sept 2006	Case/control	0–5 yrs		Clinical/healthy	381	Stool	*E. coli*	241	CLSI
Tosisa et al. (2020)	Ethiopia	Sub-Saharan Africa	Hospital	Jan – Jul 2014	Cross-sectional	5-60 months	125 m;114f	Clinical	239	Stool	*Shigella, Salmonella*	3	CLSI
Assefa and Girma (2019)	Ethiopia	Sub-Saharan Africa	Community	Apr-Jul 2016	Cross-sectional	3-60 months	179 m;243f	Clinical	422		*Shigella, Salmonella*	18	CLSI
Prabhurajeshwar et al. (2016)	India	Asia	Hospital	Jul 2013-Mar 2014	Cross-sectional	<5 yrs		Clinical	334	Stool	*Shigella*	32	CLSI
Qu et al. (2016)	China	Asia	Hospital	Apr 2010-Dec 2014	Cross-sectional	<5 yrs	1,616 m;908f	Clinical	2,524	Stool	*Shigella, Salmonella*	37	CLSI
Mandomando et al. (2009)	Mozambique	Sub-Saharan Africa	Hospital	Jul 2001-Jul 2003	Cross-sectional	<5 yrs		Clinical		Rectal swab	*Shigella, Salmonella*	109	CLSI
Ameya et al. (2018)	Ethiopia	Sub-Saharan Africa	Community	Mar-May 2017	Cross-sectional	<5 yrs		Clinical	167	Rectal swabs	*Shigella, Salmonella*	8	CLSI
Nyanga et al. (2017)	Kenya	Sub-Saharan Africa	Hospital		Cross-sectional	<5 yrs		Clinical	Males = 184	Stool	*Shigella*	14	CLSI
Dhital et al. (2017)	Nepal	Asia	Hospital	Jan-Dec 2014	Cross-sectional	<5 yrs		Clinical	717	Stool	*Shigella*	15	CLSI
Mulatu et al. (2014)	Ethiopia	Sub-Saharan Africa	Hospital	Jun -Oct 2011	Cross-sectional	<5 yrs	81 m;77f	Clinical	158	Stool/rectal swabs	*Shigella, Campylobacter, Salmonella*	11	CLSI
Sousa et al. (2013)	Brazil	Central/South America	Hospital	Mar 2004-Mar 2005	Prospective	1–48 months	83 m;74f	Clinical	157	Stool	*Shigella*	17	CLSI
Farahani et al. (2018)	Iran	North Africa/Middle East	Hospital	2012–2016	Cross-sectional	1–10 yrs		Clinical	5,300	Stool	*Shigella, Salmonella*	185	CLSI
Abebe et al. (2018)	Ethiopia	Sub-Saharan Africa	Hospital	Jun – Sept 2017	Cross-sectional	<5 yrs	101 m;103f	Clinical	204	Stool	*Shigella, Salmonella*	17	CLSI
Beyene and Tasew (2014)	Ethiopia	Sub-Saharan Africa	Hospital	Mar-Nov 2012	Cross-sectional	0–10 yrs		Clinical	260	Stool/rectal swabs	*Shigella*	6	CLSI
Beatty et al. (2009)	Kenya	Sub-Saharan Africa	Hospital	Oct 2001-Oct 2003	Cross-sectional	<5 yrs		Clinical	2,550	Stool	*Shigella, Campylobacter, Salmonella*	116	CLSI
Nunes et al. (2012)	Brazil	Central/South America	Hospital	Jan 2004 – Aug 2007	Case/control	<5 yrs		Clinical/healthy	250	Stool	*Shigella*	26	CLSI
Parajuli et al. (2017)	Nepal	Asia	Hospital	Jul-16	Case/control	6–30 months	1 m;1f	Clinical	2	Stool	*Shigella*	2	CLSI
Rahouma et al. (2011)	Libya	North Africa/Middle East	Hospital	Feb – Oct 2008	Cross-sectional	5 years		Clinical	239	Stool	*Salmonella*	19	CLSI
Bhattarai et al. (2020)	Nepal	Asia	Hospital	Nov 2017 -Apr 2018	Cross-sectional	<5 yrs	207 m;96f	Clinical	303	Stool	*Campylobacter*	172	EUCAST
Lengerh et al. (2013)	Ethiopia	Sub-Saharan Africa	Hospital	Oct 2011-Mar 2012	Cross-sectional	1 mo-5 yrs	144 m;141f	Clinical	285	Stool	*Campylobacter*	44	CLSI
Zaidi et al. (2012)	Mexico	North America	Hosp/Community	2003–2006	Cross-sectional	≤5 yrs		Clinical/healthy	2042	Stool	*Campylobacter*	105	CLSI

AST, Antimicrobial Susceptibility Testing; CLSI, Clinical and Laboratory Standards Institute; EUCAST, European Committee of Antimicrobial Susceptibility Testing.

*Health status (clinical): sick children either in the community or visiting hospital after acquiring a community infection.

The 64 selected publications included observational studies comprising 50 cross-sectional, 11 case–control, and 3 cohort studies. The antimicrobials tested against these bacteria were clinically relevant and included AMP, AMC, TET, SXT, CIP, NA, CN, CRO, and chloramphenicol (C). Only two publications examined all four bacterial species (Bodhidatta et al., 2010; Webale et al., 2020), while seven publications examined three of the four species (Moyo et al., 2011; Mekonnen et al., 2017; Ansari et al., 2012; HaiLing et al., 2017; Sang et al., 2012; Mulatu et al., 2014; Beatty et al., 2009). In addition, nine publications examined two bacterial species each (Jafari et al., 2009; Mandomando et al., 2007; Tosisa et al., 2020; Assefa and Girma, 2019; Qu et al., 2016; Mandomando et al., 2009; Ameya et al., 2018; Farahani et al., 2018; Abebe et al., 2018), while the remaining 46 publications examined only one pathogen (Singh et al., 2019; Anvikar et al., 2008; Mahdavi Broujerdi et al., 2018; Huang et al., 2015; Nakhjavani et al., 2013; Saleem et al., 2020; Snehaa et al., 2020; Araque and Labrador, 2018; Al-Saadi et al., 2018; Zheng et al., 2016; Seidman et al., 2009; Islam et al., 2019; Amin et al., 2018; Adugna et al., 2015; GebreSilasie et al., 2018; Mahmoudi-aznaveh et al., 2017; Younas et al., 2016; Stoesser et al., 2014; Vilchez et al., 2014; Giani et al., 2018; Monira et al., 2017; Naderi et al., 2016; Dias et al., 2016; Gonzales et al., 2013; Garcia et al., 2011; El Metwally et al., 2007; Souza et al., 2009; Hetzer et al., 2019; Dyar et al., 2012; Isendahl et al., 2012; Najibi et al., 2012; Sahoo et al., 2012; Amaya et al., 2011; Prabhurajeshwar et al., 2016; Nyanga et al., 2017; Dhital et al., 2017; Sousa et al., 2013; Beyene and Tasew, 2014; Nunes et al., 2012; Parajuli et al., 2017; Rahouma et al., 2011; Bhattarai et al., 2020; Lengerh et al., 2013; Zaidi et al., 2012; Singh et al., 2019; Ferjani et al., 2018). A comprehensive overview of meta-analysis results is presented in Supplementary Table 2.

### 3.1 Escherichia coli

Our meta-analysis identified high levels of *E. coli* resistance to key antibiotics, with notable regional variations. Resistance to AMP was the highest overall, with a pooled proportion of 71%, reaching 89% in North Africa/Middle East. Substantial resistance to SXT (56%) and TET (51%) was observed globally, while resistance to CIP (10%) and CRO (8%) remained low but showed regional hotspots, particularly in North Africa/Middle East and Asia.

**Figure 3:**
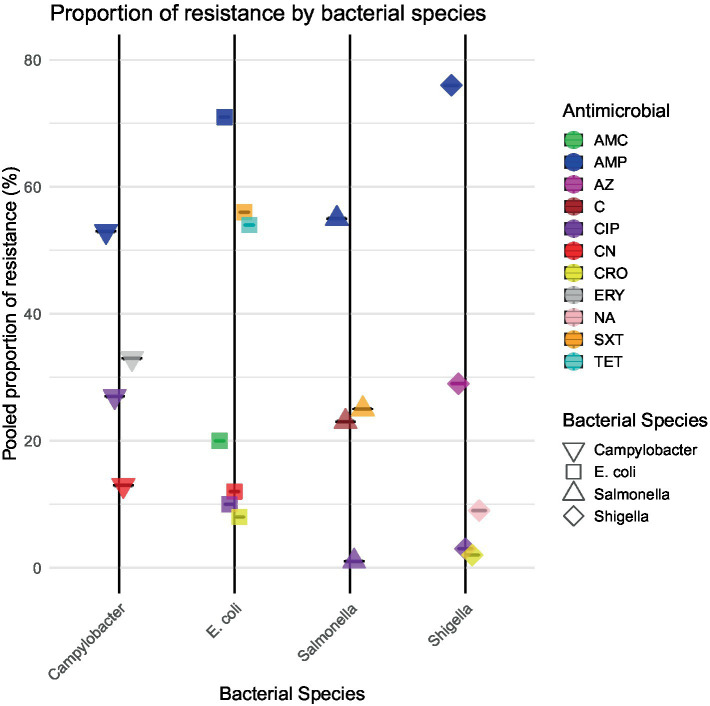
Pooled proportion of resistance of enteric bacteria against different antibiotics.

There were 32 studies with a total of 4,425 *E. coli* isolates that reported resistance to AMP. The overall pooled proportion of AMP resistance was 71%, which increased to 75% after the removal of 11 outlier studies (Figure 3).

**Figure 4:**
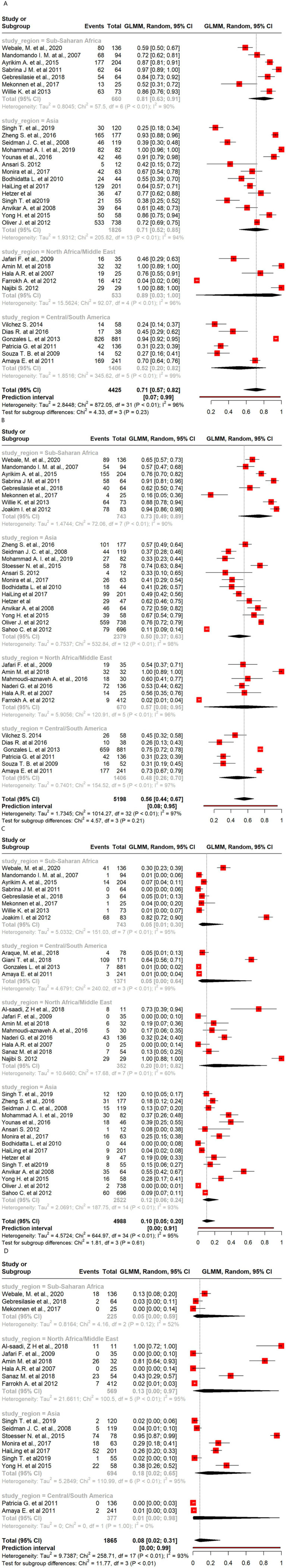
Forest plot of *E. coli* antimicrobial resistance across regions: **(A)** ampicillin; **(B)** sulfamethoxazole/trimethoprim; **(C)** ciprofloxacin; and **(D)** ceftriaxone.

Regional subgroup analyses revealed a pooled AMP resistance proportion of 89% for North Africa/Middle East, 81% for sub-Saharan Africa, 71% for Asia, and 52% for Central and South America (Figure 4A). There were 33 studies with a total of 5,198 *E. coli* isolates studied for resistance to SXT. The pooled SXT resistance proportion was 56%, which decreased to 54% after the removal of 14 outlier studies. Regional subgroup analyses revealed a pooled SXT resistance proportion of 73% for sub-Saharan Africa, 57% for North Africa/Middle East, 50% for Asia, and 48% for Central and South America (Figure 4B). Although CIP resistance was lower overall (10%), it was notably higher in North Africa/Middle East (20%) than in other regions, emphasizing the need for targeted interventions in these areas (Figure 4C). There were 18 studies with a total of 1,865 *E. coli* isolates studied for resistance to CRO. The pooled CRO resistance proportion was 8%, which decreased to 4% after the removal of three outlier studies. The pooled CRO resistance proportion was 18% in Asia, 13% in North Africa/Middle East, 5% in sub-Saharan Africa, and 1% in Central and South America (Figure 4D). A total of 21 studies investigated 2,080 *E. coli* isolates studied for resistance to AMC, reporting a pooled resistance proportion of 20%, which decreased to 17% after 10 outliers were removed. Regional subgroup analysis revealed moderate resistance to AMC, with the highest prevalence in North Africa/Middle East (51%) and the lowest in Asia (7%). Detailed statistics are provided in Supplementary Figure 1A. CN resistance was investigated by 33 studies covering 3,111 *E. coli* isolates. The pooled CN resistance proportion was 12%, which decreased to 10% after the removal of 13 outliers. Subgroup analysis showed a CN resistance of 22% among Asian isolates, while CN resistance of *E. coli* isolates from sub-Saharan Africa, North Africa/Middle East, and Central and South America was 18, 8, and 2%, respectively (Supplementary Figure 1B). There were 28 studies with a total of 4,658 *E. coli* isolates investigated for TET resistance. The pooled TET resistance proportion was 51%, which increased to 54% after the removal of nine outliers. Regional subgroup analysis revealed that *E. coli* showed a higher TET resistance in Asia (56%) than in North Africa/Middle East (51%), sub-Saharan Africa (49%), and Central and South America (39%) (Supplementary Figure 1C).

### 3.2 Shigella

Meta-analysis of *Shigella* isolates revealed large regional differences in resistance to the tested antibiotics. Resistance to AMP was highest overall with a pooled estimate of 76%, with the highest proportion in North Africa/Middle East (85%) followed by sub-Saharan Africa (79%). Resistance to CIP, CRO, and NA was low overall (<10%), but higher in specific regions, such as Asia for CIP (28%) and NA (53%).

**Figure 5:**
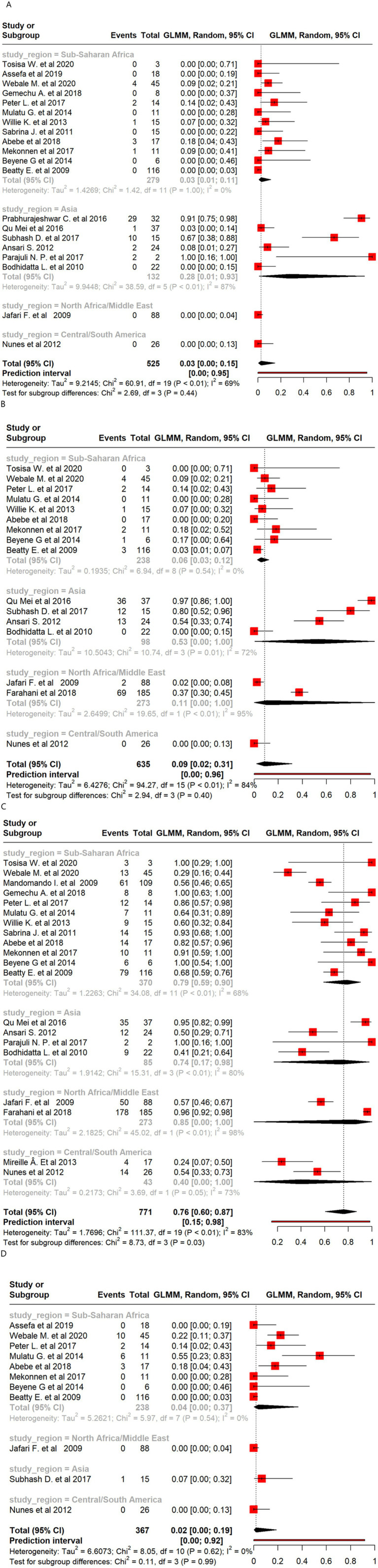
Forest plot of *Shigella* antimicrobial resistance across regions: **(A)** ciprofloxacin; **(B)** nalidixic acid; **(C)** ampicillin; and **(D)** ceftriaxone.

There were 20 studies with a total of 525 *Shigella* isolates that reported resistance to CIP. The overall pooled proportion of CIP resistance was 3% (Figure 3), which decreased to 2% after the removal of three outlier studies (Figure 3). Regional subgroup AMR analysis showed a CIP resistance of 28% in Asia and 3% in sub-Saharan Africa (Figure 5A). There were 16 studies that analyzed 635 *Shigella* isolates for resistance to NA. The overall pooled NA resistance proportion was 9%, which decreased to 5% when three outliers were removed (Supplementary Table 2). Regional subgroup analysis showed higher resistance estimates for Asian isolates (53%) than for those from North Africa/Middle East (11%) and sub-Saharan Africa (6%) (Figure 5B). There were 20 studies that analyzed 771 isolates for AMP resistance. The pooled estimate proportion for AMP resistance was 76%, which remained the same after three outliers were removed. Regional subgroup analysis showed a pooled AMP resistance proportion of 85% for North Africa/Middle East, 79% for sub-Saharan Africa, and 74% for Asia (Figure 5C). Of the 367 isolates from 11 studies that analyzed CRO resistance, a pooled resistance proportion of 2% was observed, which decreased to 1% after one outlier was removed. Of the 238 isolates investigated from eight studies in sub-Saharan Africa, the pooled proportion for CRO resistance was 4% (Figure 5D). Three studies analyzed 39 isolates showing AZM resistance, resulting in an overall resistance proportion of 29%. No outlier was removed since none was detected as shown in Supplementary Table 2.

### 3.3 Salmonella

Meta-analysis of resistance shown by *Salmonella* revealed moderate levels of resistance to the antibiotics and some regional differences. Resistance was highest for AMP (55%) and SXT (25%), with sub-Saharan Africa showing higher resistance than Asia in both cases. C resistance was also more prevalent in sub-Saharan Africa than in Asia. CIP resistance was minimal overall (1%), with no resistance observed in sub-Saharan Africa.

**Figure 6:**
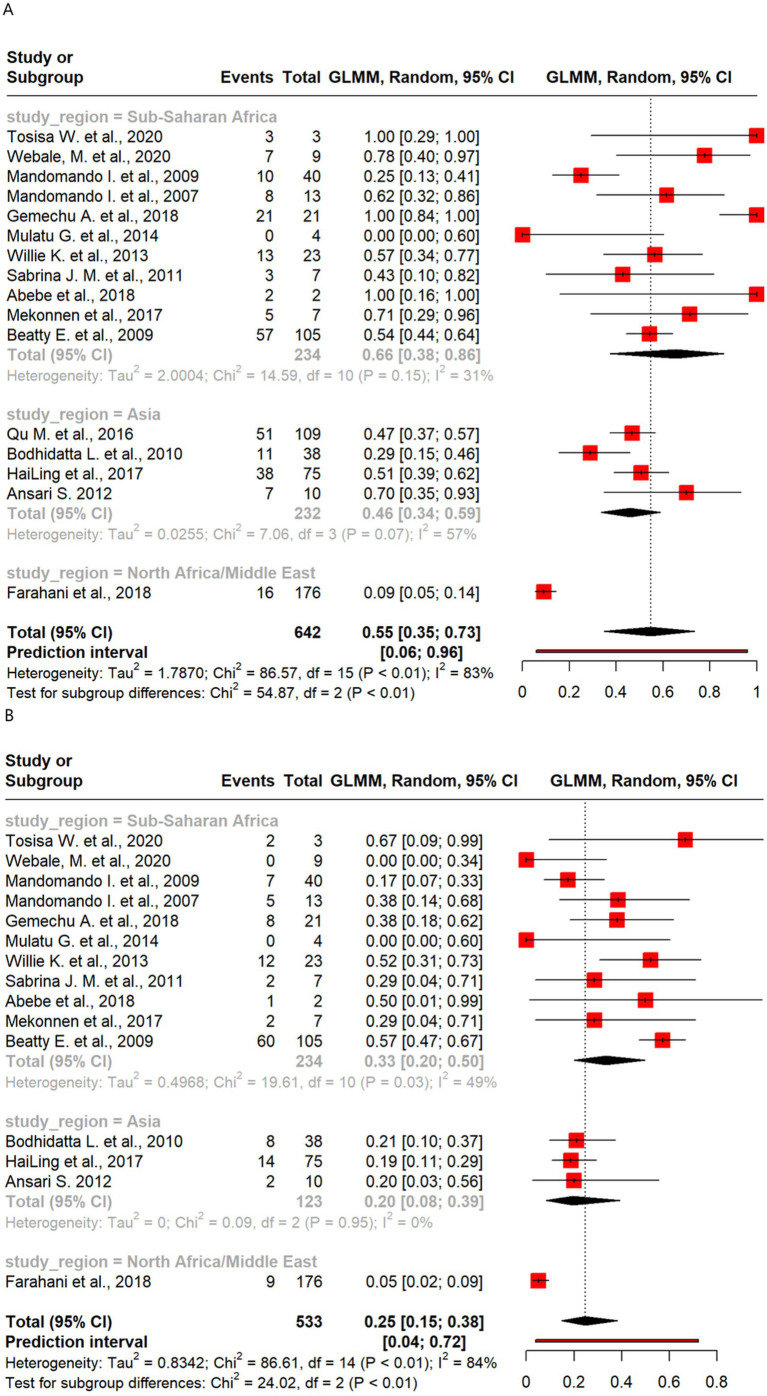
Forest plot of *Salmonella* antimicrobial resistance across regions: **(A)** ampicillin and **(B)** sulfamethoxazole/trimethoprim.

There were 16 studies that analyzed 642 *Salmonella* isolates for AMP resistance. The overall pooled proportion of AMP resistance was 55% (Figure 3), which increased to 58% after the removal of one outlier study (Figure 3). Regional subgroup analysis showed that sub-Saharan Africa had a higher pooled AMP resistance proportion than Asia (66% vs. 46%) (Figure 6A). Of the 533 isolates from 15 studies that analyzed resistance to SXT, a pooled resistance proportion of 25% was reported, which increased to 26% after the removal of two outlier studies. Regional subgroup analysis showed that sub-Saharan Africa had higher SXT resistance than Asia (33% vs. 20%) (Figure 6B). Of the 457 isolates from 15 studies that analyzed C resistance, a pooled resistance proportion of 23% was reported, which decreased to 21% after one outlier was removed (Supplementary Figure 2A). Regional subgroup analysis showed that sub-Saharan Africa had higher C resistance than Asia (28% vs. 15%). The pooled proportion of CIP resistance was 1% from 15 studies that analyzed 461 isolates. When six outliers were removed, 100% CIP susceptibility was found, as shown in Supplementary Table 2. Regional subgroup analysis showed 2% resistance in Asian isolates and 100% susceptibility among sub-Saharan African isolates (Supplementary Figure 2B).

### 3.4 Campylobacter

Meta-analysis of resistance shown by *Campylobacter* to the antibiotics highlighted notable regional differences, with higher ERY resistance observed in sub-Saharan Africa (44%) than in Asia (37%). CIP resistance was more prevalent in Asia (57%) than in sub-Saharan Africa (9%), while AMP resistance was similar across regions. CN resistance was higher in sub-Saharan Africa (33%) than in Asia (13%).

**Figure 7:**
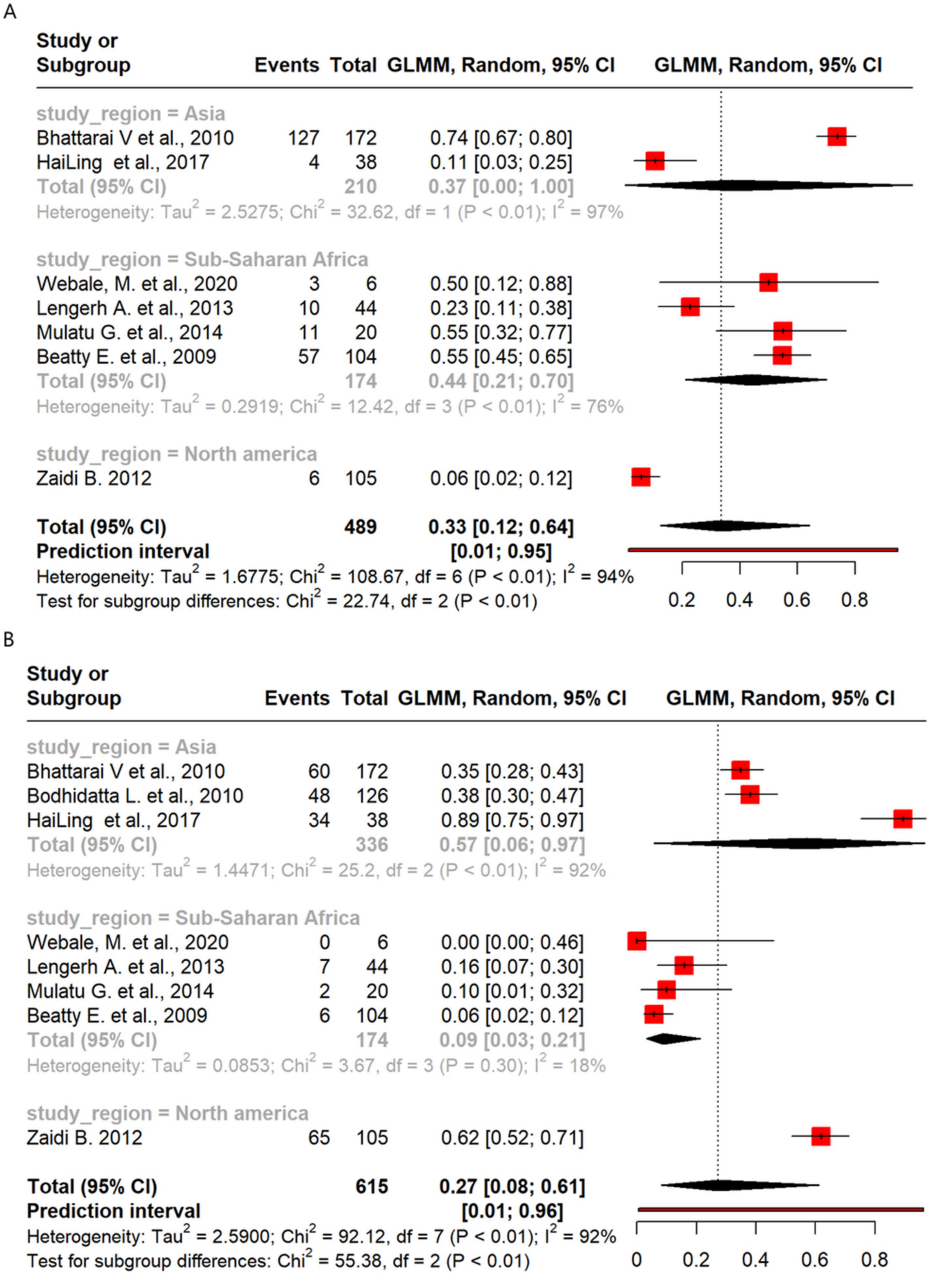
Forest plot of *Campylobacter* antimicrobial resistance across regions: **(A)** erythromycin and **(B)** ciprofloxacin.

Of the 489 isolates from seven studies that analyzed ERY resistance, a pooled resistance proportion of 33% was reported, which increased to 35% after the removal of two outlier studies. Studies from sub-Saharan Africa reported higher ERY resistance than those from Asia (44% vs. 37%) (Figure 7A). Eight studies covering 615 isolates that analyzed resistance to CIP reported a pooled resistance estimate of 27%, which decreased to 21% after the removal of one outlier study. Subgroup analysis showed higher resistance in Asia (57%) than in sub-Saharan Africa (9%) (Figures 3, 7B). There were five studies with 368 *Campylobacter* isolates that analyzed AMP resistance, which reported a pooled AMP resistance proportion of 53%, with no outlier removal. AMP resistance among Asian isolates was 58%, while that among sub-Saharan African isolates was 51% (Supplementary Figure 3A). The pooled proportion of CN resistance across the regions was 13% out of the 489 isolates investigated across seven studies, without outlier removal, with sub-Saharan Africa showing higher resistance than Asia (33% vs. 13%) (Supplementary Figure 3B).

In summary, the analysis revealed moderate (*I*^2^ > 50%) to high level (*I*^2^ > 75%) of heterogeneity between the results, with no evidence of small study effects or publication bias overall (Supplementary Table 3 and Supplementary Figure 4).

## 4 Discussion

This meta-analysis illustrates the level of pooled AMR in key bacterial enteric pathogens, which may lead to potential treatment failures of certain antimicrobials (Murray et al., 2022).

### 4.1 Escherichia coli

Our analysis of the 64 studies from LMICs showed that enteric *E. coli* were resistant to critically important antimicrobials at rates ranging from 8 to 71%. The pooled CRO resistance of 8% shows that these isolates could be Extended-spectrum beta lactamase (ESBL) producers. CRO is a third-generation cephalosporin and therefore infections with this third-generation cephalosporin (3GC)-resistant pathogens would render treatment with AMP or AMC ineffective (Abayneh et al., 2018). CRO is classified as Highest Priority Critically Important Antimicrobial (HPCIA) by WHO, and the resistance of *E. coli* to this 3GC poses a significant human health challenge (World Health Organization, 2019). Although it is not clear why *E. coli* shows higher CRO resistance in Asia than in other regions, the use of CRO as an alternative therapy in the treatment of diarrhea where other antibiotics fail has been reported in some developing Asian countries (Gu et al., 2015). This increase in resistance may be associated with the global increase in antibiotic consumption, a 65% rise between 2000 and 2015 (Klein et al., 2018). Notably, four out of six countries with the highest antibiotic consumption rates were LMICs (namely Tunisia, Algeria, Romania, and Turkey). Furthermore, overall antibiotic consumption has increased by 114% in LMICs, led by India, Pakistan, and China. The higher consumption of broad-spectrum penicillins, cephalosporins, quinolones, and macrolides by LMICs than by HICs could contribute to the increased development of resistance, including against antibiotics like CRO (Klein et al., 2018; Wieters et al., 2024). Higher CRO resistance, as reported in clinical *E. coli*, could therefore be associated with increased usage in clinical settings (Gelaw et al., 2022). ESBL-encoding genes are mostly plasmid borne and circulate in bacterial clones by horizontal transfer. ESBL-E not only circulates among community-onset and hospital infections but has also been reported in more than 60% of healthy children and adults with minimal antimicrobial exposure (Bartoloni et al., 2009). Therefore, fecal *E. coli* is a useful indicator of the spread of community-acquired AMR genes (Woerther et al., 2013; Zhang et al., 2015; Hijazi et al., 2016; Rolain, 2013; World Health Organization, 2020; United Nations Environment Programme, 2023).

Resistance to AMP among *E. coli* isolates ranged from 89% in North Africa/Middle East, 81% in sub-Saharan Africa, 71% in Asia, to 62% in Central and South America. This high level of AMP resistance is consistent with reports from various studies conducted in South Africa and Peru investigating AMP resistance in diarrheagenic *E. coli* (DEC) isolates from affected children (Omolajaiye et al., 2020; Ochoa et al., 2009). The lower AMP resistance observed in Central and South America could be due to prioritizing nutritional therapy and considering antibiotic use only in the treatment of persistent diarrhea (Dias et al., 2016). However, the 93% AMP resistance observed among Bolivian *E. coli* isolates was associated with inappropriate antimicrobial use (Gonzales et al., 2013). The AMP resistance in this study (like AMC) is concerning since this is the only WHO-recommended pediatric oral antibiotic for outpatient treatment of bacterial infections without access to hospital-based care. However, AMC is not the best option for the treatment of diarrhea as it is largely an oral formulation quickly absorbed in the gut (Okomo et al., 2019). These results on the resistance of *E. coli* to AMP are consistent with those reported in the review of commensal *E. coli* isolates collected from healthy community participants of 0–77 years of age in LMICs, indicating that AMR pathogens in healthy people may be acting as AMR reservoirs (Nji et al., 2021). Regional differences in AMP resistance shown by high between-region heterogeneity (*I*^2^ > 75%) were statistically significant (*p* < 0.01), which is reflective of high variability in sample sizes, number of isolates, and resistance prevalence across the included studies.

Resistance patterns across regions showed reduced sensitivity to SXT (56% resistance proportion), which is one of the recommended treatment choices for ESBL-E (Figure 4B). This finding was consistent with the SXT resistance proportions of *E. coli* reported in a West African review of patients attending a hospital outpatient department (Bernabé et al., 2017). Since SXT is largely used in the treatment of diarrhea, the SXT resistance reported poses a significant health concern in humans (Guarino et al., 2018). The higher SXT resistance reported in Africa than in other regions could be associated with its prophylactic use to reduce morbidity and mortality among HIV-positive patients (Morpeth et al., 2008; Said et al., 2022). TET resistance (54%) is of particular interest since TET is contraindicated in children (Hetzer et al., 2019); therefore, TET resistance in humans may be due to zoonotic transfer from animals and/or co-selection or cross resistance, e.g., through efflux pumps (Nji et al., 2021).

### 4.2 Shigella

Shigellosis, which is typically self-limiting, sometimes requires antimicrobial therapy to avoid complications, reduce dysenteric discharge, and stop prolonged fecal shedding. CIP, a fluoroquinolone like NA, is a high-priority critically important antibiotic recommended as the first-line treatment of shigellosis, especially in adult patients (WHO, 2005a). In our study, a pooled CIP resistance proportion of 3% out of 525 isolates was identified. Regional subgroup analysis revealed that *Shigella* isolates from Asia were more resistant to CIP (28% out of 132 isolates) than those from other regions, which ranged from 0 to 3%. The study by The et al. (2016) found that all CIP-resistant *Shigella* they isolated belonged to one clade, with Asia being the likely primary source. This review confirms that Asian *Shigella* is more likely to be CIP resistant. This may be due to specific genetic characteristics associated with efflux-pump-mediated fluoroquinolone resistance observed in *S. flexneri* (Azmi et al., 2014). This also reflects the global trend in *Shigella* resistance with the emergence of extensively drug-resistant (XDR) *Shigella sonnei* reported in clinical samples in Europe, where CIP resistance is documented at 42.3%, suggesting global spread (Lefèvre et al., 2023). Other fluoroquinolone-resistant *Shigella* depict a similar trend as shown by NA-resistant *Shigella* from Asia in this study (Figure 5B). This observed quinolone resistance may not be directly associated with use since quinolones are not prescribed to children but widely used in veterinary medicine. Thus, fluoroquinolone resistance reported in this review could be spreading to children from older people or foods of animal origin (Hijazi et al., 2016; González and Araque, 2013; Clermont et al., 2000). Due to the emerging CIP resistance, WHO recommends amidinopenicillins (pivmecillinam) or CRO as second-line therapies for CIP-resistant shigellosis (WHO, 2005a; Azmi et al., 2014). CRO efficacy in *Shigella* infection treatment is shown by the low pooled resistance proportion in this review (2% out of 367 isolates). However, inappropriate use (dose, frequency, or duration) in more than 60% of patients that ever get CRO in Africa could further lead to the emergence and spread of resistance (Bishaw et al., 2021). Due to AMP resistance across regions, as also depicted in this study (76% out of 771 isolates), WHO highlighted AMP as one of the antimicrobials that is inappropriate for shigellosis treatment (WHO, 2005a; Williams and Berkley, 2018).

### 4.3 Salmonella

*Salmonella* is an archetypal zoonotic Enterobacterale implicated in self-limiting diarrheal disease in humans, with children aged ≤5 years particularly affected (Park et al., 2021). SXT, C, and AMP are the most used antibiotics in the empiric treatment of salmonellosis. The resistance proportion of *Salmonella* to SXT, C, and AMP was mostly higher in sub-Saharan African studies (33, 28, and 66%) than in Asian studies (20, 15, and 46%), respectively. The huge increase in antimicrobial consumption in Africa and Asia could be the reason for this reported resistance (Murray et al., 2022; Browne et al., 2021). This could result in the use of other antimicrobials that are not specifically indicated for salmonellosis treatment, such as CIP and CRO. Park et al. (2021) reported the spread of CIP- and CRO-resistant *Salmonella typhimurium* ST313 and Enteritidis ST11 in several countries in Africa using genotypic methods. They attributed the resistance to the growing use of these antimicrobials to treat febrile illnesses in Africa. However, our study showed low resistance to CIP (1% out of 461 isolates) and CRO (0%), which may be because the review focused on studies reporting lab-based phenotypic AMR profiles of the isolates as opposed to complete AMR genotypes.

### 4.4 Campylobacter

In this meta-analysis, eight studies analyzed the AMR of *Campylobacter* from 615 isolates. ERY and CN resistance proportions were higher in sub-Saharan African isolates than in Asian isolates, whereas AMP and CIP resistance proportions were higher in Asian than in sub-Saharan African isolates. In studies that isolated mixed CESS isolates, *Campylobacter* spp. was the most predominant etiology of resistance to commonly prescribed antimicrobials (Mulatu et al., 2014). Hlashwayo et al. (2021) investigated the resistance of *Campylobacter* isolated from all age groups in Africa and reported that its resistance to ERY was 43% (vs. 33% in this study), TET was 43% (vs. 43%), CIP was 16% (vs. 27%), NA was 36% (vs. 35%), and CN was 35% (vs. 13%). These resistance patterns are consistent with ours, and inappropriate use of antimicrobials in LMICs could partly explain this phenomenon. TET resistance observed in our study may have included zoonotic transfer from animals and/or co-selection or cross-resistance through efflux pumps and mobile genetic elements, such as transposons (Nji et al., 2021). *Campylobacter* is commonly isolated from poultry, and the use of CIP and other quinolones in poultry farming is widely practiced (Coulidiaty et al., 2021). Therefore, CIP resistance in these community *Campylobacter* isolates from children could be due to zoonotic transfer.

The highest AMR proportion among these CESS infections was observed against AMP, with a pooled resistance proportion higher than 50%. This suggests that antimicrobials recommended for empiric treatment in children are losing their efficacy. AMP and CIP resistance proportions are far higher than those of clinical isolates reported in HICs that practice judicious antimicrobial use (AMP 8.2%, CIP 2.1%) but consistent with AMR clinical isolates reported in Ghana (Asare et al., 2022).

This systematic review demonstrates that CRO is largely effective in treating enteric bacterial infections in children. However, the emerging CRO resistance observed in the community isolates of *E. coli* and *Shigella* could explain the level of non-efficacy of AMP and AMC across CESS. This could as well be associated with inappropriate CRO use in hospital settings in Africa (Clermont et al., 2000). These resistance patterns are comparable with those reported in hospital settings (Dias et al., 2016; Ochoa et al., 2009). The evident misuse of CRO in African hospitals and CRO and CIP prescriptions for febrile illnesses in LMICs could further increase resistance (Wieters et al., 2024; Bishaw et al., 2021; Park et al., 2021). SXT resistance presented in our study is consistent with those reported in clinical cases of DEC and other enteric bacteria (Bernabé et al., 2017). Although quinolones are not prescribed for pediatric acute gastroenteritis cases, CIP and NA resistance may have resulted due to transmission from older relatives or via foods of animal origin due to common CIP usage in veterinary practice; the same holds for C and TET resistance, where co-selection is a possible phenomenon (Ananchaipattana et al., 2014). The evidence from this review underpins the need to conduct interventions not only in hospital settings but also in community. The current policies with regard to outpatient management of diarrhea in children should focus on appropriate antimicrobial stewardship and alternative interventions such as water, sanitation and hygiene (WASH) programs that could be effective in curbing the emergence and spread of AMR in LMICs (Simiyu et al., 2020; Awiti et al., 2019; Simiyu et al., 2023; Fuhrmeister et al., 2023). However, these approaches should be context specific since they are likely to be influenced by socioeconomic status in LMICs.

Previously published reviews have focused on HAI infections (Ayobami et al., 2022; Allegranzi et al., 2011; Le Doare et al., 2015). They have reported the highest prevalence of *Klebsiella pneumonia* resistance to AMP (93.8%) and CN (68.8%), with overall Enterobacteriaceae resistance to AMP at 79.6%. Our findings of *E. coli* AMP resistance across regions (52–89%) are consistent with these reports, highlighting the importance of community surveillance in predicting bacteria with AMR and mitigating the future spread of resistance to clinical settings.

The regional disparities in pooled AMR prevalence estimates observed in this study can be attributed to several factors, including differences in healthcare access, antibiotic usage policies, and regulatory frameworks (Iskandar et al., 2021). Regions with limited healthcare infrastructure, such as parts of sub-Saharan Africa, often experience higher AMR due to over-the-counter access to antibiotics, poor adherence to treatment regimens, and limited diagnostic capacity (Omulo et al., 2015; Omulo et al., 2017). The high resistance proportions observed in North Africa/Middle East and Asia may be associated with increased antibiotic use over the past decade. These findings highlight the need for region-specific interventions, including improving healthcare infrastructure, strengthening antibiotic policies, and raising public awareness to mitigate AMR effectively.

Our study assessed pooled AMR prevalence in community-acquired enteric bacteria, namely *E. coli*, *Salmonella*, *Shigella*, and *Campylobacter*. While our focus was on pooled AMR prevalence from community-acquired infections, understanding colonization prevalence, especially for *E. coli*, is crucial. The ubiquitous presence of *E. coli* in the gastrointestinal tract underscores the importance of colonization prevalence data. Unlike *Salmonella*, *Shigella*, and *Campylobacter*, high colonization prevalence of *E. coli* may not directly correlate with AMR prevalence. However, it can significantly determine the risk of AMR development and transmission.

### 4.5 Limitations

We observed a high variance in resistance proportions across the individual study estimates. Heterogeneity between studies and between regions was in most cases *I*^2^ ≥ 75%. This high heterogeneity is likely attributable to differences in study design, setting, sampling design, and bacterial isolation and identification methods, as well as variations in AST methods. Since studies conducted after 2005 were included, it is highly likely that some studies used outdated Clinical and Laboratory Standards Institute (CLSI) and European Committee of Antimicrobial Susceptibility Testing (EUCAST) breakpoints. This could introduce bias as we were unable to reinterpret or adjust resistance data based on the latest breakpoints due to the reporting of results as the percentage or the number of resistant isolates rather than disk inhibition zones or minimum inhibitory concentration values. We included only studies that used laboratory-based phenotypic AST methods whose data are interpretable using CLSI or EUCAST, thus losing out on more robust methods that reported AMR genotypes. This might have limited the robustness of the findings by overlooking the genetic mechanisms of resistance. Furthermore, the 64 studies included in this systematic review and meta-analysis came from 23 countries (against a total of 82 LMICs) and thus may not be representative of all WHO geographical regions. Regions with limited research infrastructure or fewer published studies may be underrepresented, leading to gaps in data coverage and potential underestimation of resistance in these areas.

Studies published before 2005 were excluded to ensure alignment with WHO’s intensified efforts to address AMR as a global public health threat that started around this period. Excluding studies with older data or different methodologies helps maintain consistency and relevance in the reported findings as older studies may not reflect current testing standards or resistance patterns. However, this exclusion may limit historical context and trends, potentially underestimating the evolution of resistance over time.

To address these constraints future research should focus on harmonizing AST methodologies, incorporating genotypic data for a more comprehensive understanding of resistance mechanisms, and ensuring broader geographical representation by including studies from underrepresented regions. Expanding databases to include data reported in various formats could also help mitigate publication bias and enhance the generalizability of findings.

## 5 Conclusion

The present systematic review provides evidence of the prevalence of resistant enteric bacteria in children. It provides evidence that these CESS pathogens are increasingly becoming insensitive to clinically important antimicrobials. Regional differences in resistance patterns among these community isolates underpin the need to strengthen local and regional AMR surveillance systems to understand these reported differences. This would then inform clinical practice and the development of appropriate stewardship measures. Since the research and development pipeline of novel antimicrobials is lacking, there is an urgent need for the management of antimicrobials to prolong their use and explore alternative therapies such as vaccine development and use that reduce febrile illnesses, thus negating the need for antibiotic prescription.

## Data Availability

The original contributions presented in the study are included in the article/Supplementary material, further inquiries can be directed to the corresponding author.
