# Supplementary material for: Antimicrobial resistance in community-acquired enteric pathogens among children aged ≤ 10-years in low-and middle-income countries: a systematic review and meta-analysis
Source: Front Microbiol. 2025 Apr 28;16:1539160. doi: 10.3389/fmicb.2025.1539160 (PMC12066647; doi:10.3389/fmicb.2025.1539160)
Supplement: Supplementary file 1 [file Data_Sheet_1.zip › Supplementary File 1. Search syntax.docx]

Supplementary File 1. Search syntax for the six electronic databases

1. PUBMED: first searched 5^th^ August 2020, revised search 20^th^ September 2024

| Concept | Search | Query | Hits |
| --- | --- | --- | --- |
| 1. Children | #1 | Search: "Child"[Mesh] | 66485 |
|  | #2 | Search: "Infant"[Mesh] OR "Infant, Newborn"[Mesh] | 34049 |
|  | #4 | Search: Babies | 35625 |
|  | #4 | Search: Children | 89237 |
|  | #5 | Search: Neonate | 19027 |
|  | #6 | Search: (#1 OR #2 OR #3 OR #4 OR #5) | 100,745 |
| 1. Antibiotic resistance | #7 | Search: "Drug Resistance, Microbial"[Mesh] **OR "Drug Resistance"[Mesh] OR "Drug Resistance, Bacterial"[Mesh]** | 12,944 |
|  | #8 | Search: “Drug resistance” | 7,931 |
|  | #9 | Search: “Antibiotic resistance” | 532 |
|  | #10 | Search: “Antibacterial resistance” | 20 |
|  | #11 | Search: “Antimicrobial resistance” | 395 |
|  | #12 | Search: (#7 OR #8 OR #9 OR #10 OR #11) | 14035 |
| 1. Low- and middle-income countries | #13 | Search: ("Developing Countries" or "Developing country" or "Low- and Middle-income countries" or "low-income country" or "Low-income countries" or LIC or "middle-income country" or "Middle-income countries" or "middle income country" or "low income country" or MIC or LMIC or "Northern Africa" or "Africa South of the Sahara" or "Central Africa" or "Eastern Africa" or "Southern Africa" or "Western Africa" or Asia or "South Asia" or "Southern Asia" or "Southeastern Asia" or Afghanistan or Algeria or Angola or Albania or Argentina or Armenia or "American Samoa" or Azerbaijan or Bangladesh or Benin or Bhutan or Botswana or "Burkina Faso" or Burundi or Bulgaria or "Bosnia and Herzegovina" or Belarus or Belize or Bolivia or Brazil or Cambodia or China or Cameroon or "Cape Verde" or "Cabo Verde" or "Central African Republic" or Chad or Comoros or Congo or "Congo Republic" or "Cote d'Ivoire" or Columbia or "Costa Rica" or Cuba or Djibouti or "Democratic Republic of the Congo" or Dominica or "Dominican Republic" or Timor-Leste or Ecuador or Egypt or Eritrea or Ethiopia or "Equatorial Guinea" or Eswatini or Fiji or "Federated States of Micronesia" or Gabon or Gambia or Ghana or Guinea or Guinea-Bissau or Georgia or Grenada or Guatemala or Guyana or Honduras or Haiti or India or Indonesia or Iran or Iraq or Jamaica or Jordan or Kazakhstan or Kenya or "Kyrgyz Republic" or Kiribati or "Lao PDR" or Lebanon or Lesotho or Liberia or Libya or "St. Lucia" or Madagascar or Malawi or Malaysia or Maldives or Mali or Mauritania or Mauritius or Morocco or Mozambique or Myanmar or Moldova or Mexico or "Marshall Islands" or Montenegro or Mongolia or Namibia or Nauru or Nepal or Niger or Nigeria or Nicaragua or North Macedonia or "North Korea" or Pakistan or "Papua New Guinea" or Paraguay or Philippines or Peru or Romania or "Russian Federation" or Rwanda or "Saint Vincent" or "Sao Tome and Principe" or Senegal or Seychelles or Sierra Leone or "Sri Lanka" or Somalia or "South Africa" or "Solomon Islands" or "El Salvador" or Serbia or "South Sudan" or Sudan or Suriname or "Syrian Arab Republic" or Swaziland or Tanzania or Tajikistan or Turkmenistan or Tonga or Turkey or Tuvalu or Thailand or Togo or Tunisia or Uganda or Ukraine or Uzbekistan or Vietnam or Vanuatu or Venezuela or Samoa or Kosovo or "West Bank and Gaza" or "Yemen Republic" or Zambia or Zimbabwe) | 174,770 |
| Final Terms Combined | #14 | (#6 AND #12 AND #13) | **827** |

1. COCHRANE LIBRARY searched 4^th^ August 2020, revised search 20^th^ September 2024

| Concept |  | Key words | Hits |
| --- | --- | --- | --- |
| 1. Children | #1 | MeSH descriptor: [Child] explode all trees | 29385 |
|  | #2 | MeSH descriptor: [Infant] explode all trees | 15798 |
|  | #3 | MeSH descriptor: [Infant, Newborn] explode all trees | 15666 |
|  | #4 | Babies | 5510 |
|  | #5 | Children | 161042 |
|  | #6 | Neonate | 2626 |
| 1. Antibiotic resistance | #7 | MeSH descriptor: [Drug Resistance, Microbial] explode all trees | 2265 |
|  | #8 | Antibiotic resistan* | 4888 |
|  | #9 | Drug resistan* | 39604 |
|  | #10 | Antimicrobial resistan* | 2771 |
|  | #11 | Antibacterial resistan* | 2685 |
| 1. Low- and Middle-income countries | #12 | ("Developing Countries" or "Developing country" or "Low- and Middle-income countries" or "low-income country" or "Low-income countries" or LIC or "middle-income country" or "Middle-income countries" or "middle income country" or "low income country" or MIC or LMIC or "Northern Africa" or "Africa South of the Sahara" or "Central Africa" or "Eastern Africa" or "Southern Africa" or "Western Africa" or Asia or "South Asia" or "Southern Asia" or "Southeastern Asia" or Afghanistan or Algeria or Angola or Albania or Argentina or Armenia or "American Samoa" or Azerbaijan or Bangladesh or Benin or Bhutan or Botswana or "Burkina Faso" or Burundi or Bulgaria or "Bosnia and Herzegovina" or Belarus or Belize or Bolivia or Brazil or Cambodia or China or Cameroon or "Cape Verde" or "Cabo Verde" or "Central African Republic" or Chad or Comoros or Congo or "Congo Republic" or "Cote d'Ivoire" or Columbia or "Costa Rica" or Cuba or Djibouti or "Democratic Republic of the Congo" or Dominica or "Dominican Republic" or Timor-Leste or Ecuador or Egypt or Eritrea or Ethiopia or "Equatorial Guinea" or Eswatini or Fiji or "Federated States of Micronesia" or Gabon or Gambia or Ghana or Guinea or Guinea-Bissau or Georgia or Grenada or Guatemala or Guyana or Honduras or Haiti or India or Indonesia or Iran or Iraq or Jamaica or Jordan or Kazakhstan or Kenya or "Kyrgyz Republic" or Kiribati or "Lao PDR" or Lebanon or Lesotho or Liberia or Libya or "St. Lucia" or Madagascar or Malawi or Malaysia or Maldives or Mali or Mauritania or Mauritius or Morocco or Mozambique or Myanmar or Moldova or Mexico or "Marshall Islands" or Montenegro or Mongolia or Namibia or Nauru or Nepal or Niger or Nigeria or Nicaragua or North Macedonia or "North Korea" or Pakistan or "Papua New Guinea" or Paraguay or Philippines or Peru or Romania or "Russian Federation" or Rwanda or "Saint Vincent" or "Sao Tome and Principe" or Senegal or Seychelles or Sierra Leone or "Sri Lanka" or Somalia or "South Africa" or "Solomon Islands" or "El Salvador" or Serbia or "South Sudan" or Sudan or Suriname or "Syrian Arab Republic" or Swaziland or Tanzania or Tajikistan or Turkmenistan or Tonga or Turkey or Tuvalu or Thailand or Togo or Tunisia or Uganda or Ukraine or Uzbekistan or Vietnam or Vanuatu or Venezuela or Samoa or Kosovo or "West Bank and Gaza" or "Yemen Republic" or Zambia or Zimbabwe) | 197780 |
| Final Combined | #13 | ((#1 or #2 or #3 or #4 or #5 or #6) and (#7 or #8 or #9 or #10 or #11) and (#12)) | 1162 |

1. CABI searched 4^TH^ August 2020, revised search 21^st^ September 2024

| Concept |  | Key words | Hits |
| --- | --- | --- | --- |
| 1. Children | #1 | (((child*) OR (infant) OR ("infant, newborn") OR (babies) OR (neonate)) AND yr:[2005 TO 2020]) AND ( ((item-type:(( "Journal article" ) )) )) | 128550 |
| 1. Antibiotic resistance | #2 | (("drug resistance, microbial") OR ("drug resistance, bacterial") OR ("drug resistan*") OR ("antibiotic resistan*") OR ("antibacterial resistan*") OR ("antimicrobial resistan*")) AND yr:[2005 TO 2020]) AND ( ((item-type:(( "Journal article" ) )) )) | 38368 |
|  | #3 | (("antibiotic resistance") OR ("drug resistance") AND yr:[2005 TO 2020]) AND ( ((item-type:(( "Journal article" ) )) )) | 36856 |
|  | #4 | #2 OR #3 | 38368 |
|  |  | (#1) AND (#2) | 9545 |
| 1. Low- and Middle-income countries | #5 | Refined by:  (geographic-location:(( "Africa South of Sahara" OR "India" OR "Brazil" OR "Turkey" OR "Taiwan" OR "developing countries" OR "Thailand" OR "Guangdong" OR "Korea Republic" OR "Vietnam" OR "Mexico" OR "Tunisia" OR "Burkina Faso" OR "Karnataka" OR "Chongqing" OR "Sao Paulo" OR "Colombia" OR "Indonesia" OR "Hebei" OR "Uttar Pradesh" OR "Papua New Guinea" OR "Zimbabwe" OR "China" OR "Iran" OR "Ethiopia" OR "Pakistan" OR "Egypt" OR "Ghana" OR "Malawi" OR "Shanghai" OR "Tamil Nadu" OR "Hubei" OR "Cambodia" OR "Mozambique" OR "Jiangsu" OR "Senegal" OR "Romania" OR "Malaysia" OR "Congo Democratic Republic" OR "Henan" OR "South Africa" OR "Nigeria" OR "Tanzania" OR "Kenya" OR "Uganda" OR "Zhejiang" OR "Africa" OR "Beijing" OR "Bangladesh" OR "Delhi" OR "Peru" OR "Nepal" OR "Maharashtra" OR "Argentina" OR "Cameroon" OR "Russia" OR "Sichuan" OR "South East Asia" OR "Zambia" OR "Mali" ) )) )) | 4561 |
|  | #6 | "developing countr*" yr:[2005 TO 2020] |  |
|  | #7 | "middle income countr*" yr:[2005 TO 2020] |  |
|  | #8 | "low income countr*" yr:[2005 TO 2020] |  |
|  | #9 | 6 OR 7 OR 8 |  |
|  | #10 | Refined by (#5 OR #9) | 4561 |
| Final terms Combined | #11 | (#1 AND #2 AND #5) | 4561 |

1. Embase searched 14^th^ July 2020, revised search 22^nd^ September 2024

Note: forward slash (/) after term denotes a MESH term (or equivalent)

The ‘mp. …’ shows the areas that the database automatically searched for within the paper it’s categorisation.

| Concept |  | Key words | Hits |
| --- | --- | --- | --- |
| 1. Children | 1 | (child*OR infant* or newborn or neonat* or babies or children)  ab,kw,ti,tw. | 1903784 |
| 1. Antibiotic resistance | 2 | ("Antibiotic Resistan*" or "Antimicrobial Resistan*" or "Drug Resistan*" or "Antibacterial Resistan*")  ab,kw,ti,tw. | 199920 |
|  | 3 | antibiotic resistance/ or drug resistance/ | 256163 |
|  | 4 | 2 or 3 | 362017 |
| 1. Low- and middle-income countries | 5 | ("Developing Countries" or "Low- and Middle-income countries" or "low-income country" or "Low-income countries" or LIC or "middle-income country" or "Middle-income countries" or MIC or LMIC or "Northern Africa" or "Africa South of the Sahara" or "Central Africa" or "Eastern Africa" or "Southern Africa" or "Western Africa" or Asia or "South Asia" or "Southern Asia" or "Southeastern Asia" or Afghanistan or Algeria or Angola or Albania or Argentina or Armenia or "American Samoa" or Azerbaijan or Bangladesh or Benin or Bhutan or Botswana or "Burkina Faso" or Burundi or Bulgaria or "Bosnia and Herzegovina" or Belarus or Belize or Bolivia or Brazil or Cambodia or China or Cameroon or "Cape Verde" or "Cabo Verde" or "Central African Republic" or Chad or Comoros or Congo or "Congo Republic" or "Cote d'Ivoire" or Columbia or "Costa Rica" or Cuba or Djibouti or "Democratic Republic of the Congo" or Dominica or "Dominican Republic" or Timor-Leste or Ecuador or Egypt or Eritrea or Ethiopia or "Equatorial Guinea" or Eswatini or Fiji or "Federated States of Micronesia" or Gabon or Gambia or Ghana or Guinea or Guinea-Bissau or Georgia or Grenada or Guatemala or Guyana or Honduras or Haiti or India or Indonesia or Iran or Iraq or Jamaica or Jordan or Kazakhstan or Kenya or "Kyrgyz Republic" or Kiribati or "Lao PDR" or Lebanon or Lesotho or Liberia or Libya or "St. Lucia" or Madagascar or Malawi or Malaysia or Maldives or Mali or Mauritania or Mauritius or Morocco or Mozambique or Myanmar or Moldova or Mexico or "Marshall Islands" or Montenegro or Mongolia or Namibia or Nauru or Nepal or Niger or Nigeria or Nicaragua or North Macedonia or "North Korea" or Pakistan or "Papua New Guinea" or Paraguay or Philippines or Peru or Romania or "Russian Federation" or Rwanda or "Saint Vincent" or "Sao Tome and Principe" or Senegal or Seychelles or Sierra Leone or "Sri Lanka" or Somalia or "South Africa" or "Solomon Islands" or "El Salvador" or Serbia or "South Sudan" or Sudan or Suriname or "Syrian Arab Republic" or Swaziland or Tanzania or Tajikistan or Turkmenistan or Tonga or Turkey or Tuvalu or Thailand or Togo or Tunisia or Uganda or Ukraine or Uzbekistan or Vietnam or Vanuatu or Venezuela or Samoa or Kosovo or "West Bank and Gaza" or "Yemen Republic" or Zambia or Zimbabwe)  .ab,kw,ti,tw. | 1539605 |
|  | 6 | exp developing country/ | 95947 |
|  | 7 | exp middle income country/ | 8723 |
|  | 8 | exp low income country/ | 6066 |
|  | 9 | 5 or 6 or 7 or 8 | 1584609 |
| FINAL terms combined |  | 1 and 4 and 9 | 4950 |

1. Medline searched 14^th^ July 2020, revised search 22^nd^ September 2024

Note: forward slash (/) after term denotes a MESH term (or equivalent)

The ‘mp. …’ shows the areas that the database automatically searched for within the paper it’s categorisation.

| Concept |  | Key words | Hits |
| --- | --- | --- | --- |
| 1. Children | 1 | (child*OR infant* or newborn or neonat* or babies or children)  ab,kw,ti,tw. | 1228738 |
| 1. Antibiotic resistance | 2 | ("Antibiotic Resistan*" or "Antimicrobial Resistan*" or "Drug Resistan*" or "Antibacterial Resistan*")  ab,kw,ti,tw. | 122751 |
|  | 3 | exp Drug Resistance, Microbial/ | 163476 |
|  | 4 | exp Drug Resistance/ | 328675 |
|  | 5 | 2 or 3 or 4 | 376958 |
| 1. Low- and middle-income countries | 6 | ("Developing Countries" or "Low- and Middle-income countries" or "low-income country" or "Low-income countries" or LIC or "middle-income country" or "Middle-income countries" or MIC or LMIC or "Northern Africa" or "Africa South of the Sahara" or "Central Africa" or "Eastern Africa" or "Southern Africa" or "Western Africa" or Asia or "South Asia" or "Southern Asia" or "Southeastern Asia" or Afghanistan or Algeria or Angola or Albania or Argentina or Armenia or "American Samoa" or Azerbaijan or Bangladesh or Benin or Bhutan or Botswana or "Burkina Faso" or Burundi or Bulgaria or "Bosnia and Herzegovina" or Belarus or Belize or Bolivia or Brazil or Cambodia or China or Cameroon or "Cape Verde" or "Cabo Verde" or "Central African Republic" or Chad or Comoros or Congo or "Congo Republic" or "Cote d'Ivoire" or Columbia or "Costa Rica" or Cuba or Djibouti or "Democratic Republic of the Congo" or Dominica or "Dominican Republic" or Timor-Leste or Ecuador or Egypt or Eritrea or Ethiopia or "Equatorial Guinea" or Eswatini or Fiji or "Federated States of Micronesia" or Gabon or Gambia or Ghana or Guinea or Guinea-Bissau or Georgia or Grenada or Guatemala or Guyana or Honduras or Haiti or India or Indonesia or Iran or Iraq or Jamaica or Jordan or Kazakhstan or Kenya or "Kyrgyz Republic" or Kiribati or "Lao PDR" or Lebanon or Lesotho or Liberia or Libya or "St. Lucia" or Madagascar or Malawi or Malaysia or Maldives or Mali or Mauritania or Mauritius or Morocco or Mozambique or Myanmar or Moldova or Mexico or "Marshall Islands" or Montenegro or Mongolia or Namibia or Nauru or Nepal or Niger or Nigeria or Nicaragua or North Macedonia or "North Korea" or Pakistan or "Papua New Guinea" or Paraguay or Philippines or Peru or Romania or "Russian Federation" or Rwanda or "Saint Vincent" or "Sao Tome and Principe" or Senegal or Seychelles or Sierra Leone or "Sri Lanka" or Somalia or "South Africa" or "Solomon Islands" or "El Salvador" or Serbia or "South Sudan" or Sudan or Suriname or "Syrian Arab Republic" or Swaziland or Tanzania or Tajikistan or Turkmenistan or Tonga or Turkey or Tuvalu or Thailand or Togo or Tunisia or Uganda or Ukraine or Uzbekistan or Vietnam or Vanuatu or Venezuela or Samoa or Kosovo or "West Bank and Gaza" or "Yemen Republic" or Zambia or Zimbabwe)  .ab,kw,ti,tw. | 969924 |
|  | 7 | exp Developing Countries/ | 74663 |
|  | 8 | 6 or 7 | 993860 |
| FINAL terms combined |  | 1 and 5 and 8 | 3747 |

1. Web of Science, searched 14^th^ July 2020, revised search 22^nd^ September 2024

| Concept |  | Key words | Hits |
| --- | --- | --- | --- |
| 1. Children | 1 | **TOPIC:**  (child*OR infant* or newborn or neonat* or babies or children) | 1959682 |
| 1. Antibiotic resistance | 2 | **TOPIC:**  ("Antibiotic Resistan*" or "Antimicrobial Resistan*" or "Drug Resistan*" or "Antibacterial Resistan*") | 184611 |
| 1. Low- and middle-income countries | 3 | TOPIC: ("Developing Countries" or "Low- and Middle-income countries" or "low-income country" or "Low-income countries" or LIC or "middle-income country" or "Middle-income countries" or MIC or LMIC or "Northern Africa" or "Africa South of the Sahara" or "Central Africa" or "Eastern Africa" or "Southern Africa" or "Western Africa" or Asia or "South Asia" or "Southern Asia" or "Southeastern Asia" or Afghanistan or Algeria or Angola or Albania or Argentina or Armenia or "American Samoa" or Azerbaijan or Bangladesh or Benin or Bhutan or Botswana or "Burkina Faso" or Burundi or Bulgaria or "Bosnia and Herzegovina" or Belarus or Belize or Bolivia or Brazil or Cambodia or China or Cameroon or "Cape Verde" or "Cabo Verde" or "Central African Republic" or Chad or Comoros or Congo or "Congo Republic" or "Cote d'Ivoire" or Columbia or "Costa Rica" or Cuba or Djibouti or "Democratic Republic of the Congo" or Dominica or "Dominican Republic" or Timor-Leste or Ecuador or Egypt or Eritrea or Ethiopia or "Equatorial Guinea" or Eswatini or Fiji or "Federated States of Micronesia" or Gabon or Gambia or Ghana or Guinea or Guinea-Bissau or Georgia or Grenada or Guatemala or Guyana or Honduras or Haiti or India or Indonesia or Iran or Iraq or Jamaica or Jordan or Kazakhstan or Kenya or "Kyrgyz Republic" or Kiribati or "Lao PDR" or Lebanon or Lesotho or Liberia or Libya or "St. Lucia" or Madagascar or Malawi or Malaysia or Maldives or Mali or Mauritania or Mauritius or Morocco or Mozambique or Myanmar or Moldova or Mexico or "Marshall Islands" or Montenegro or Mongolia or Namibia or Nauru or Nepal or Niger or Nigeria or Nicaragua or North Macedonia or "North Korea" or Pakistan or "Papua New Guinea" or Paraguay or Philippines or Peru or Romania or "Russian Federation" or Rwanda or "Saint Vincent" or "Sao Tome and Principe" or Senegal or Seychelles or Sierra Leone or "Sri Lanka" or Somalia or "South Africa" or "Solomon Islands" or "El Salvador" or Serbia or "South Sudan" or Sudan or Suriname or "Syrian Arab Republic" or Swaziland or Tanzania or Tajikistan or Turkmenistan or Tonga or Turkey or Tuvalu or Thailand or Togo or Tunisia or Uganda or Ukraine or Uzbekistan or Vietnam or Vanuatu or Venezuela or Samoa or Kosovo or "West Bank and Gaza" or "Yemen Republic" or Zambia or Zimbabwe) | 3061562 |
| FINAL COMBINED |  | #3 AND #2 AND #1 | 4217 |
