# Supplementary figures and images for: Antimicrobial resistance in community-acquired enteric pathogens among children aged ≤ 10-years in low-and middle-income countries: a systematic review and meta-analysis
Source: Front Microbiol. 2025 Apr 28;16:1539160. doi: 10.3389/fmicb.2025.1539160 (PMC12066647; doi:10.3389/fmicb.2025.1539160)

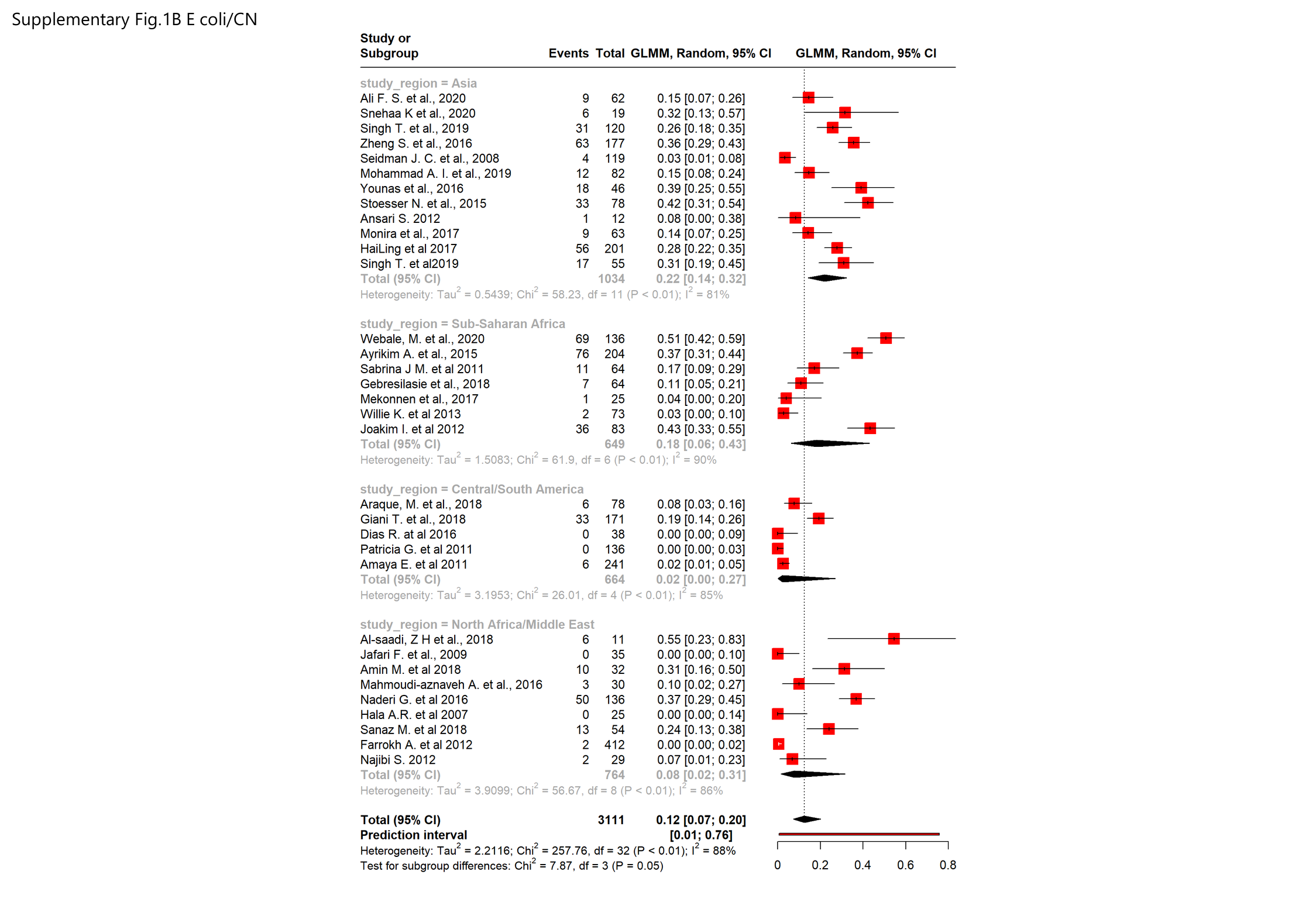

Supplement: Supplementary file 1 [file Data_Sheet_1.zip › Supplementary Figure 1B. E. coli resistance to gentamicin by subgroup.tiff]

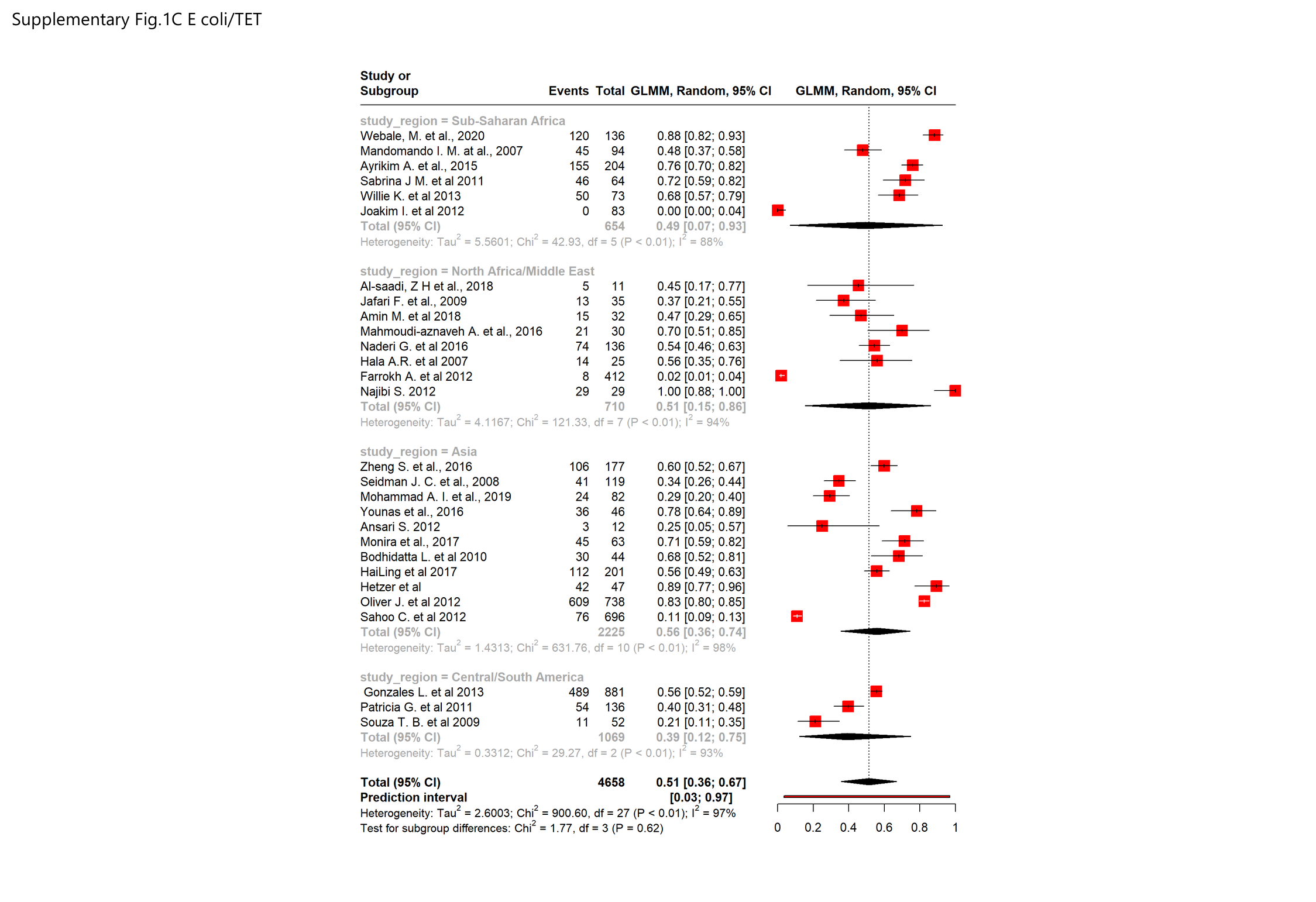

Supplement: Supplementary file 1 [file Data_Sheet_1.zip › Supplementary Figure 1C. E. coli resistance to tetracycline by subgroup.tiff]

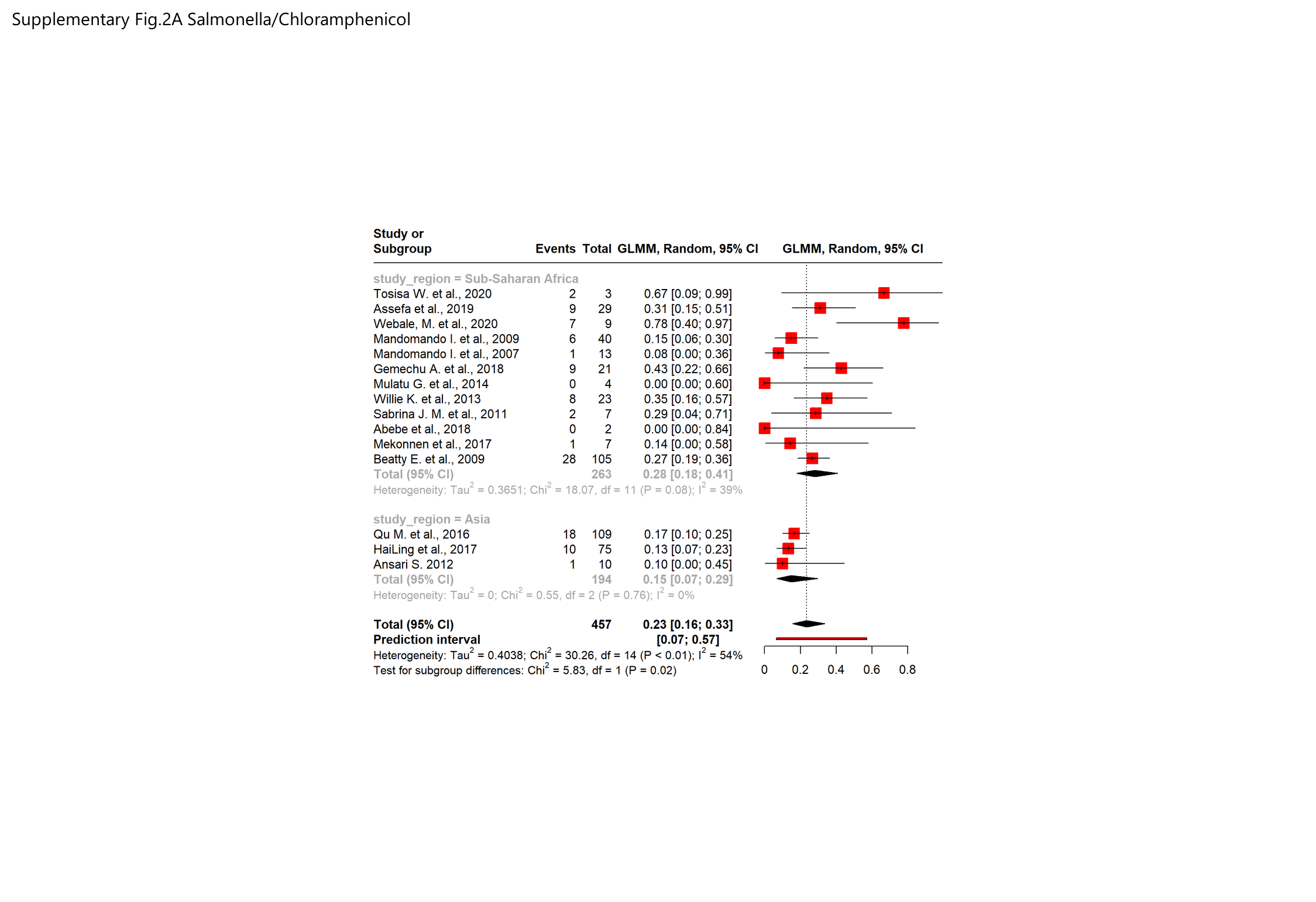

Supplement: Supplementary file 1 [file Data_Sheet_1.zip › Supplementary Figure 2A. Salmonella resistance to chloramphenicol by subgroup.tiff]

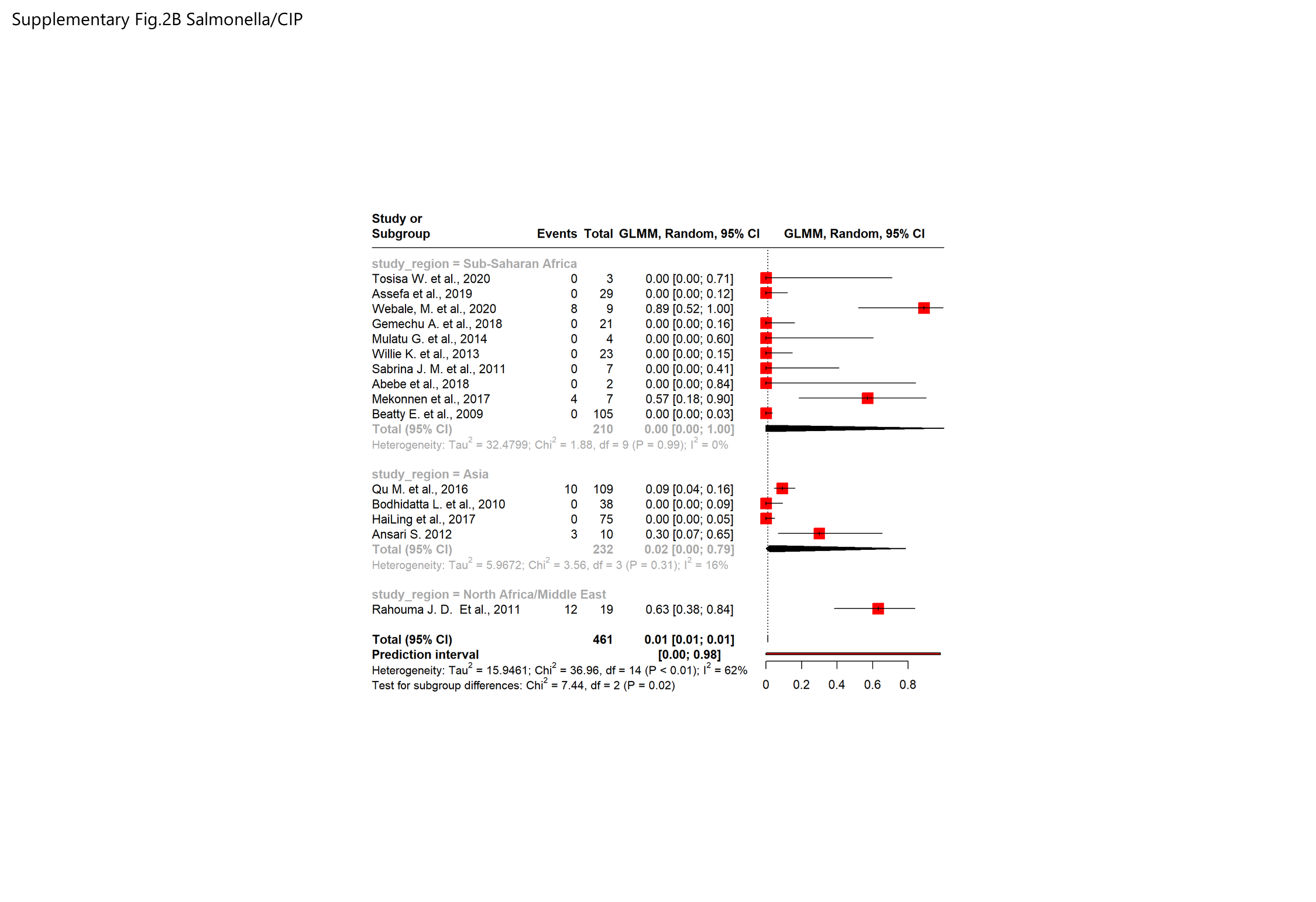

Supplement: Supplementary file 1 [file Data_Sheet_1.zip › Supplementary Figure 2B. Salmonella resistance to ciprofloxacin by subgroup.tiff]

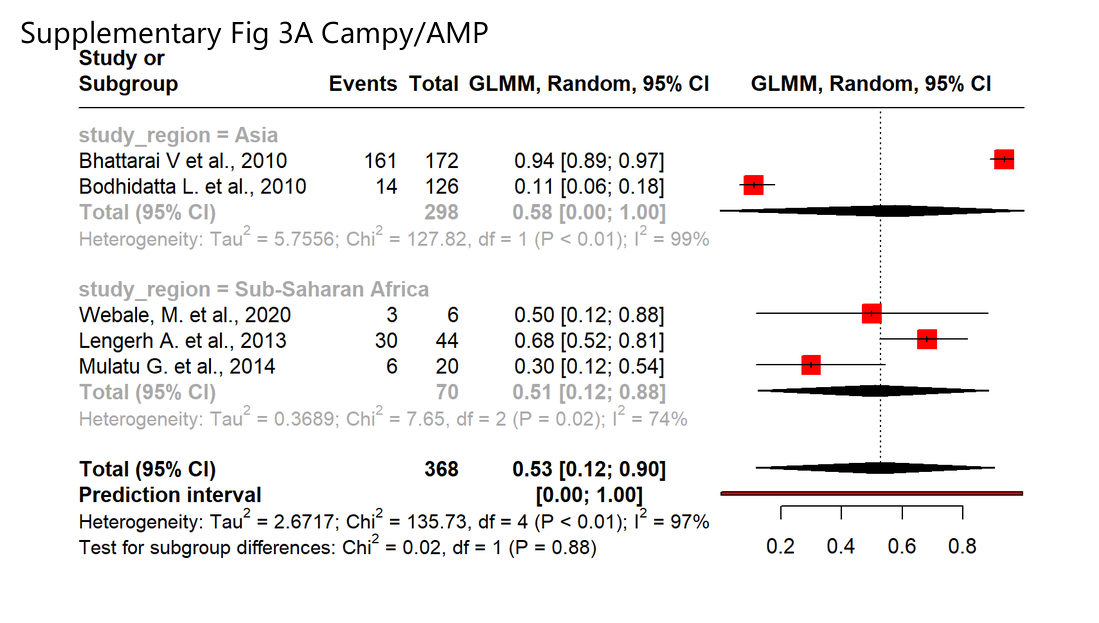

Supplement: Supplementary file 1 [file Data_Sheet_1.zip › Supplementary Figure 3A. Campylobacter resistance to ampicillin by subgroup.tiff]

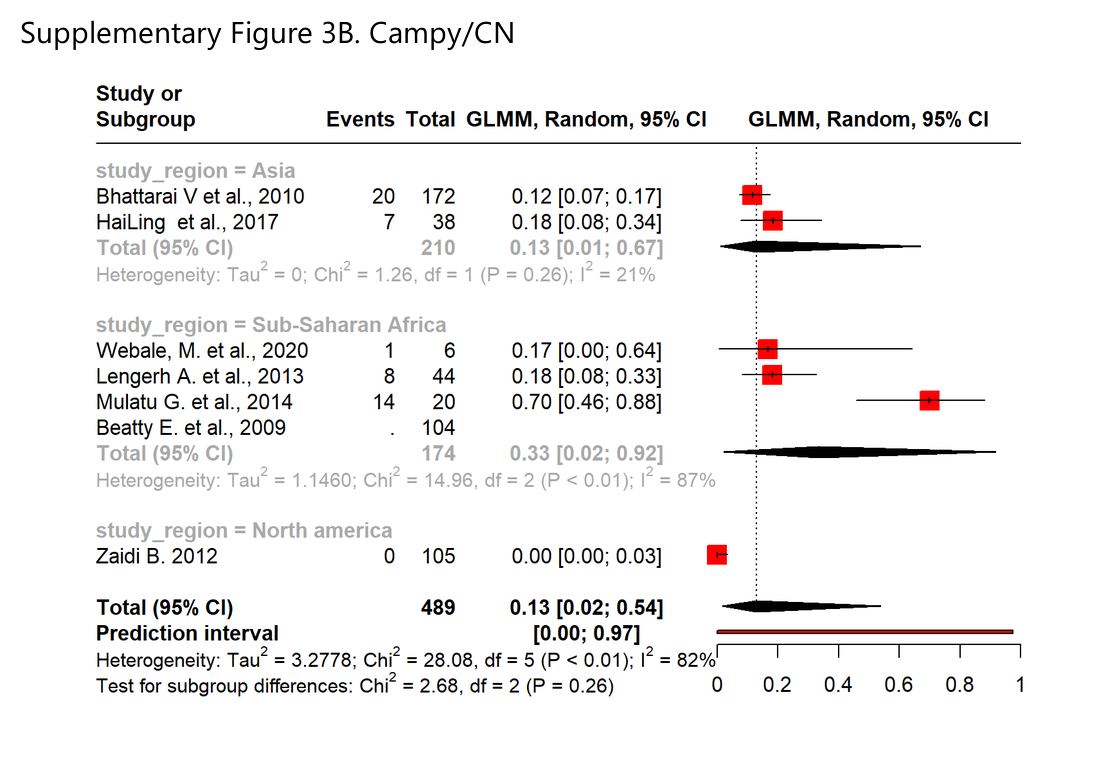

Supplement: Supplementary file 1 [file Data_Sheet_1.zip › Supplementary Figure 3B. Campylobacter resistance to gentamicin by subgroup.tiff]

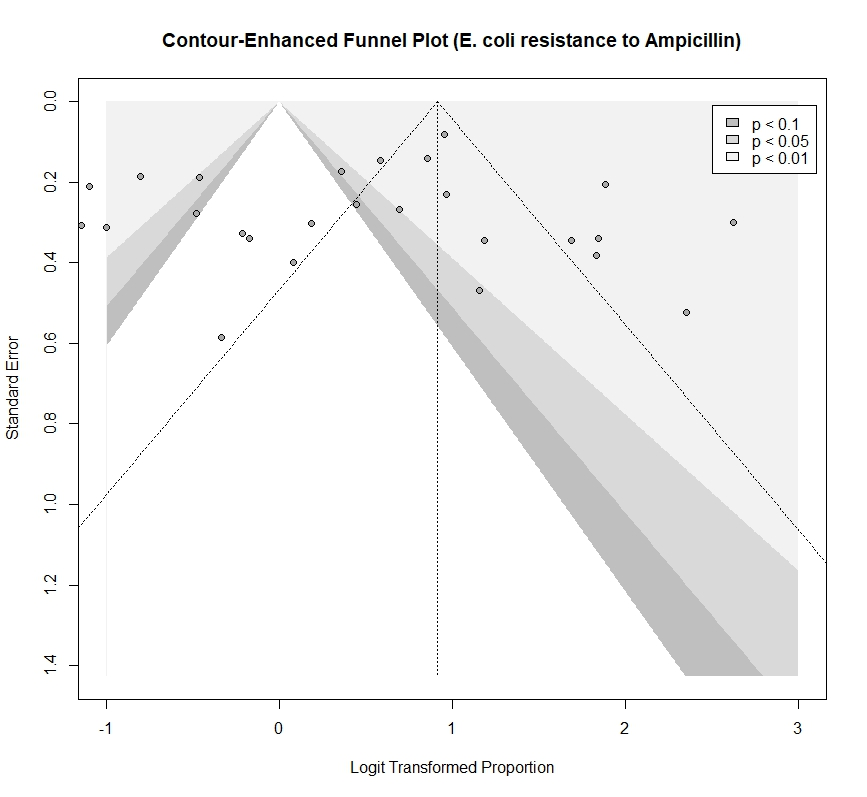

Supplement: Supplementary file 1 [file Data_Sheet_1.zip › Supplementary Figure 4. Funnel plot of publication bias analysis of E. coli resistance to ampicillin.tif]

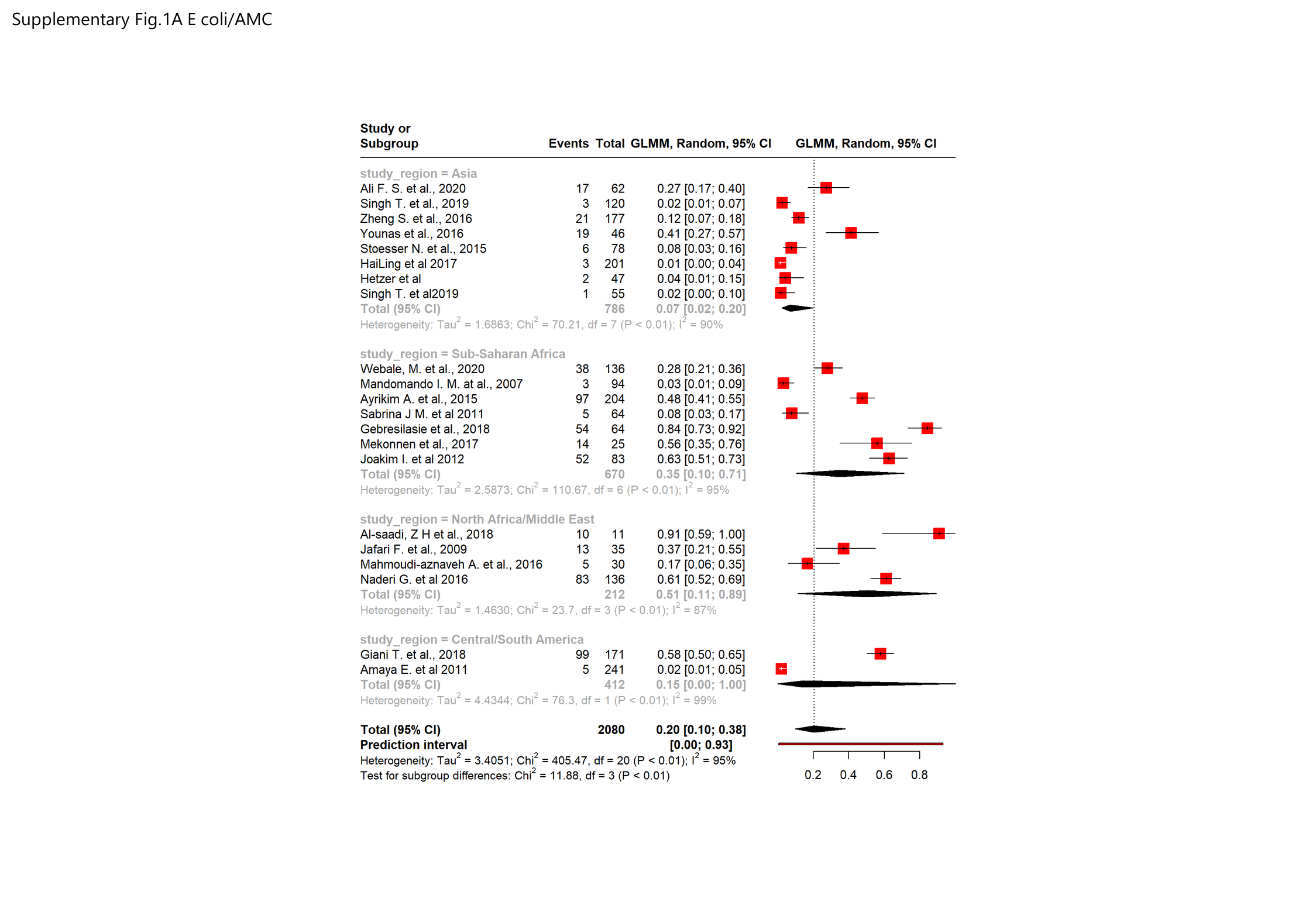

Supplement: Supplementary file 1 [file Data_Sheet_1.zip › Supplementary Figure 1A. E. coli resistance to Amoxicillin or clavulanic acid by subgroup.tiff]
